# PILRB potentiates the PI3K/AKT signaling pathway and reprograms cholesterol metabolism to drive gastric tumorigenesis and metastasis

**DOI:** 10.1038/s41419-024-07026-5

**Published:** 2024-09-03

**Authors:** Xing Wang, Yuanyuan Liu, Qiuyan Zhao, Xin Wang, Xinyi Chen, Li Hou, Shaodan Tian, Zi-Mei Peng, Xiao-Jian Han, Tao Wang, Zhen Zhang, Fang-Fang Tou, Shan Huang, Jun Rao, Lixiao Chen, Zhi Zheng

**Affiliations:** 1 Centre for Medical Research and Translation, Jiangxi Provincial People’s Hospital, The First Affiliated Hospital of Nanchang Medical College, Nanchang, 330006 China; 2 Department of Otolaryngology: Head and Neck Surgery, Shanghai General Hospital, Shanghai Jiao Tong University School of Medicine, Shanghai, 200080 China; 3 Department of Gastroenterology, Henan Provincial People’s Hospital, People’s Hospital of Zhengzhou University, Zhengzhou, Henan China; 4 Department of Hematology, The First Affiliated Hospital of Chongqing Medical University, Chongqing, 400016 China; 5 Dongzhimen Hospital, Beijing University of Chinese Medicine, Chaoyang, China; 6 Institute of Clinical Medicine, Jiangxi Provincial People’s Hospital, The First Affiliated Hospital of Nanchang Medical College, Nanchang, 330006 China; 7 Institute of Geriatrics, Jiangxi Provincial People’s Hospital, The First Affiliated Hospital of Nanchang Medical College, Nanchang, Jiangxi 330006 PR China; 8 Jiangxi Provincial People’s Hospital, The First Affiliated Hospital of Nanchang Medical College, Nanchang, 330006 China; 9 Jiangxi Cancer Hospital, The Second Affiliated Hospital of Nanchang Medical College, Jiangxi Clinical Research Center for Cancer, Nanchang, Jiangxi PR China

**Keywords:** Gastric cancer, Gastric cancer

## Abstract

Paired immunoglobin-like type 2 receptor beta (PILRB) mainly plays a crucial role in regulating innate immunity, but whether PILRB is involved in cancer is poorly understood. Here, we report that PILRB potentiates the PI3K/AKT pathway to drive gastric tumorigenesis by binding and stabilizing IRS4, which could hyperactivate the PI3K/AKT pathway. Firstly, the levels of PILRB are upregulated in human gastric cancer (GC) specimens and associated with poor prognosis in patients with GC. In addition, our data show that PILRB promotes cell proliferation, colony formation, cell migration and invasion in GC cells in vitro and in vivo. Mechanistically, PILRB recruits the deubiquitination enzymes OTUB1 to IRS4 and relieves K48-linked ubiquitination of IRS4, protecting IRS4 protein from proteasomal-mediated degradation and subsequent activation of the PI3K/AKT pathway. Importantly, the levels of PILRB are positively correlated with IRS4 in GC specimens. Meanwhile, we also found that PILRB reprogrammed cholesterol metabolism by altering ABCA1 and SCARB1 expression levels, and *PILRB*-expression confers GC cell resistance to statin treatment. Taken together, our findings illustrate that the oncogenic role of PILRB in gastric tumorigenesis, providing new insights into the regulation of PI3K/AKT signaling in GC and establishing *PILRB* as a biomarker for simvastatin therapy resistance in GC.

## Introduction

Gastric cancer (GC) is a complex and aggressive disease with heterogeneous features that accounts for a notable proportion of cancer-related deaths worldwide [1, 2]. GC is the most common adenocarcinoma, exhibiting various molecular and histologic subtypes influenced by multiple genetic and epigenetic alterations, infections, and the tumor environment [3, 4]. Although accumulating evidence suggests a recent decline in incidence due to the advancements in surgical approaches and adjuvant chemotherapy, GC continues to rank as the world’s third most common cause of cancer mortality, which is attributed to the lack of sensitive and specific early diagnostic markers [5]. Owing to its heterogeneity and complexity, GC is an aggressive disease with a poor prognosis. Patients are always diagnosed at an advanced stage, presenting with regional metastasis at the time of diagnosis [6]. Lymph node metastasis is one of the most common indicators of prognosis and recurrence in patients with GC. The number of metastatic lymph nodes negatively correlates with tumor aggressiveness and prognosis [7, 8]. Consequently, further elucidation of the molecular mechanisms regulating GC recurrence and metastasis is urgently needed to improve the prognosis of patients with GC.

The paired immunoglobulin-like type 2 receptor (PILR) belongs to the Ig superfamily, comprising the inhibitory receptor PILR alpha (PILRA) and the activating receptor PILR beta (PILRB). Both PILRA and PILRB share high similarities in their extracellular domains, suggesting that they may recognize similar ligands although their intracellular domains are different [9, 10]. PILRA has two immunoreceptor tyrosine-based inhibitory motifs (ITIM) in its intracellular domain that recruit the phosphatases SHP-1 and SHP-2 to induce an inhibitory signaling cascade, resulting in reduced calcium mobilization in the cytoplasm [11, 12]. Although PILRB contains a truncated intracellular domain without an immunoreceptor tyrosine-based activation motif (ITAM), it has a charged amino acid residue in its transmembrane region that binds to the ITAM-bearing DAP12 adapter molecule to trigger an activating signal [13]. PILRB is primarily expressed in myeloid lineages, including natural killer (NK) cells, dendritic cells, and macrophages. PILRB-knockout mice were challenged with *Toxoplasma gondii*-induced inflammation, including chronic encephalitis and inflammatory bowel disease [14, 15]. Previous studies reported that PILRB interacts with various ligands, including CD99, which appear to regulate immune cells and inflammation. NK cells and dendritic cells can be activated by cells expressing PILR ligands [14]. To the best of our knowledge, no previous studies have evaluated the expression levels of PILRB in tumor cells. Furthermore, no evidence has been shown regarding the contribution of PILRB to cancer cell progression or metastasis.

Reprogramming cholesterol metabolism has always occurred in most tumors, which promotes tumor progression and metastasis by maintaining cellular cholesterol homeostasis [16]. Cellular cholesterol homeostasis is controlled by the synthesis, influx, efflux, and metabolism [17, 18]. In cancer, homeostatic cholesterol processes can be disturbed to facilitate cancer cell’s survival and aberrant proliferation. Targeting cholesterol metabolism is a promising strategy for numerous malignancies and has been widely tested in clinics [19]. However, the benefits are modest, and this treatment is hindered by the lack of a promising biomarker to identify GC patients who may benefit from the cholesterol-lowering agents [20]. Therefore, a complete understanding of cholesterol metabolism is needed for improving the efficacy of targeting cholesterol metabolism to treat cancer. To date, the importance of PILRB in regulating reprogramming cholesterol metabolism remains to be uncovered.

In this study, we aimed to determine the expression of *PILRB* and its clinical relevance in GC. Our results revealed that ectopic PILRB expression is significantly associated with more aggressive malignancies and poorer survival outcomes in GC. In vitro and in vivo functional experiments with *PILRB* knockdown and overexpressing GC cells suggested oncogenic functions of PILRB in tumorigenicity and metastasis. Moreover, PILRB conferred resistance to AKT inhibitors in GC cells. Mechanistically, PILRB mainly relied on the hyperactivation of the PI3K/AKT signaling pathway by stabilizing IRS4. The specific molecular mechanism involves the direct interaction of PILRB with IRS4 in GC cells, subsequently inhibiting IRS4 ubiquitination/degradation through the recruitment of OTUB1. Meanwhile, we also found that *PILRB* could decrease the GC cellular cholesterol level depending on altering ABCA1 and SCARB1 expression levels and that *PILRB* confers gastric cell resistance to statins therapy. Overall, this study elucidates insight into the role of PILRB in regulating the PI3K/AKT pathway and provides a promising therapeutic strategy for GC patients with PILRB expression.

## Material and methods

### Cells

Human GC cells, including AGS, BGC823, HGC-27, MGC-803, MKN45, SGC7901, and SNU-1, and the human gastric epithelial cell line GES-1 were obtained from Thousand Sunrise (Shanghai, China). The human embryonic kidney cell line HEK293T was obtained from the China Center for Type Culture Collection (CCTCC, Shanghai, China). Cells were authenticated using short tandem repeat fingerprinting. GES-1, AGS, BGC823, HGC-27, MGC-803, MKN45, SGC7901, and SNU-1 were cultured in RPMI-1640, and HEK293T cells in Dulbecco’s Modified Eagle Medium supplemented with 10% fetal bovine serum (FBS) and 100 U/mL penicillin–streptomycin (Life Technologies, USA) and maintained at 37 °C with 5% CO_2_. We ensured that all cell lines were authenticated by STR profiling and tested for mycoplasma infection.

### Immunohistochemistry (IHC)

IHC staining was performed on paraffin-embedded GC and adjacent normal tissue sections using antibodies (Table S1). Briefly, specimens were cut into 5 μm sections and baked at 65 °C for 30 min. Sections were deparaffinized, and were submerged in antigenic retrieval buffer, and microwaved for antigenic retrieval. The endogenous peroxidase activity was quenched using 3% hydrogen peroxide in methanol. Subsequently, the sections were incubated with 1% FBS to block nonspecific binding, and finally treated with antibodies overnight at 4 °C. The following day, after washing with primary antibodies, the samples were incubated with secondary antibodies for 2 h, followed by further treatment with a streptavidin-horseradish peroxidase complex. Two independent pathologists, who were blinded to the clinicopathological outcomes, evaluated and scored the IHC staining. The final score was taken as the median, and scores below the median indicated low expression, whereas above the median suggested high expression.

### Western blotting and immunoprecipitation

Western blotting was performed according to the standard protocol. Briefly, the samples were separated by sodium dodecyl sulfate-polyacrylamide gel electrophoresis (SDS-PAGE) and blotted onto polyvinylidene fluoride membranes. Membranes were blocked by 5% fat-free milk in TBST for one hour and then incubated with the primary antibodies overnight at 4 °C. After washing three times with TBST, the membranes were incubated with secondary antibodies for an hour. Finally, the bands were detected using chemiluminescence. For co-immunoprecipitation (Co-IP) assay, cell lysates were immunoprecipitated with Protein A/G agarose plus antibodies or anti-FLAG-M2 agarose for 4–6 h at 4 °C and then washed three times with lysis buffer, boiled in 2×SDS loading buffer, separated by SDS-PAGE and analyzed by western blotting.

### RNA extraction, qRT-PCR analysis, and transcriptome sequencing analysis

Total RNA was isolated from GC cells using TRIzol reagent (Invitrogen, USA), and cDNA was synthesized with random primers using the PrimeScript^TM^ RT reagent kit (Takara) and served as a template for real-time PCR using the Bio-Rad CFX96^TM^ Real-time PCR System (Bio-Rad) according to the manufacturer’s instructions. Glyceraldehyde 3-phosphate dehydrogenase (GAPDH) was used as an internal control. The relative mRNA expression levels were determined using the 2^ΔΔCt^ method. The qRT-PCR primer sequences are listed in Supplementary Table S2. All reactions were performed in triplicates. For transcriptome sequencing analysis, total RNA was extracted from shNC- and shPILRB-AGS cells according to the protocol described in our previous study.

### Lentivirus infection for stable cells and plasmid transfection

The lentivirus used in this study was purchased from Sangon Biotech (Shanghai, China). To get a stable cell line, the lentivirus particles were directly added into GC cells and then treated with puromycin (1–3 μg/mL) for 14 days. Knockdown efficiency was validated by qRT-PCR and western blotting. All the vectors were confirmed by sequencing prior to use. The plasmids were transfected into the cells using PEI, following the manufacturer’s instructions.

### Cell proliferation and colony formation assays

For cell proliferation assay, 2 × 10^3^ cells were seeded into a 96-well plate and then incubated with CCK-8 solution for 2 h at 37 °C following the manufacturer’s protocol at indicated time points after seeding. For the colony formation assay, gastric cells were collected using 0.25% trypsin, counted, and plated in new 6-well plates at 500 cells/dish. After approximately 14 d, the cells were washed with PBS, fixed in 4% paraformaldehyde for 30 min, and stained with 1% crystal violet.

### Cell migration and invasion assay

A wound-healing assay was used to verify cell migration. Briefly, GC cells in the exponential phase of proliferation were collected and seeded in a new 6-well plate. When the cells reached 90% confluence, three separate wounds were created by mechanically scratching with a 200 μL pipet tip, moving perpendicular to the line. Next, the cells were gently washed with PBS to remove the floating cells. Images of the scratches were acquired using an inverted microscope at the indicated time points. For the migration assays, 3 × 10^4^ GC cells were harvested and seeded into 8.0 μm pore (24-well insert; 8 μm pore, BD Biosciences) inserts without Matrigel. The migration time was 48 h. For the invasion assays, a total of 3 × 10^4^ GC cells were digested and seeded into the upper chamber with the Matrigel-coated membrane (24-well insert; 8 μm pore, BD Biosciences) without FBS 1640 culture medium, while a complete culture medium with 10% FBS was used as the chemo-attractant in the lower chamber. After 24 h, invading cells were fixed in 4% paraformaldehyde and stained with 0.1% crystal violet. Finally, invading cells in five random fields of view were counted. Each experiment was conducted in triplicate, and the mean values are shown.

### MG132 or cycloheximide (CHX) treatment

GC cells were transfected with plasmid or shRNA and then were treated with MG132 (10 μM, S2619, Selleck) or CHX (100 μg/mL, S7418, Selleck) for different durations. Samples from the above cells were harvested with RIPA lysis buffer and fractionated by SDS-PAGE, followed by western blotting.

### In vivo ubiquitination assay

The cells were transfected with plasmids or shRNA and treated with MG132 for 6 h before collection. Samples from the above cells were harvested with lysis buffer and fractionated using SDS-PAGE.

### Cholesterol assay

Cellular total cholesterol levels were detected by a Total Cholesterol Assay Kit (Cell Biolabs). Briefly, cellular lipids were harvested by chloroform: 2-propanon:NP-40 (7:11:0.1) in a micro-homogenizer, and the cholesterol levels were measured according to the manufacturer’s protocols.

### In vivo tumorigenicity and metastasis models

To evaluate the tumorigenic effect of PILRB, 4- to 5-week-old female BALB/C nude mice were randomly assigned to two groups and were injected subcutaneously with PILRB-deficiency and control MKN-28 cells (2 × 10^6^ suspended in 100 μL PBS) in each mouse. Tumor size was monitored every 4 days by measuring the length and width of the tumor, where length (*L*) was the longest diameter and width (*W*) the shortest diameter. The tumor volume was calculated according to the standard formula: *V* = 1/2 × *L* × *W*^2^. At the end of this stage, the mice were euthanized, and the tumors were excised, imaged, and weighed. For the tail veil injection model, 2 × 10^6^ GC cells in 200 μL sterile PBS from different groups were injected into the median tail vein of nude mice. The mice were sacrificed 90 days after injection, and the lungs were collected, fixed in 4% PFA, embedded in paraffin, and stained with hematoxylin and eosin (H&E). The metastatic nodules in the lungs were observed under a microscope. All animal experiments were performed in accordance with the guidelines of animal experiments in the Laboratory Animal Center. Male 4–6-week-old nude mice were purchased from Cyagen (Shanghai, China) and used as xenograft models. 5 × 10^6^ cells transfected with shNC and shPILRB were suspended in 60 µl PBS and injected into the footpads of the mice. Six weeks after injection, the mice were euthanized, and popliteal lymph nodes were removed for measurement and IHC analysis.

### Statistical analysis

All sample sizes were sufficiently large to ensure proper statistical analyses, which were performed using GraphPrime software. Data were analyzed using a two-sided Student’s *t*-test or one- or two-way ANOVA according to the situation. Kaplan–Meier plots with log-rank tests were used to analyze the survival data. *P* < 0.05 was considered statistically significant (ns, no significance, * *P* < 0.05; ** *P* < 0.01; *** *P* < 0.001; **** *P* < 0.001).

## Results

### PILRB level was elevated within GC, which predicted a poor prognosis

**Fig. 1:**
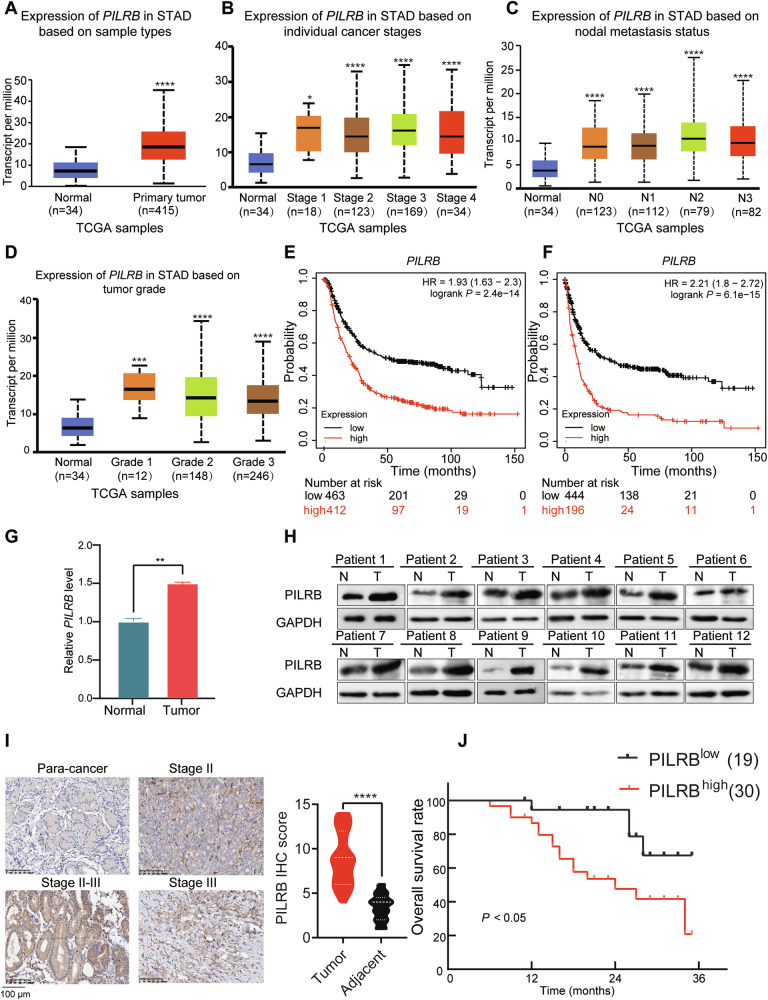
Elevated PILRB expression associated with poor clinical outcomes in GC patients. **A** TCGA database on the UALCAN website displayed that *PILRB* mRNA level was increased in stomach adenocarcinoma (STAD) tissues. **B** TCGA database on the UALCAN website revealed that PILRB expression is higher in STAD patients with advanced clinical stage compared to adjacent normal tissues. **C** TCGA database on the UALCAN website indicated that STAD patients with advanced lymph node metastasis had remarkably higher PILRB expression compared to adjacent normal tissues. **D** TCGA database on the UALCAN website illustrated that patients with high tumor grade had higher STAD expression compared to adjacent normal tissues. **E**, **F** Kaplan–Meier estimates of overall survival (**E**) and PF (**F**) in STAD patients by different PILRB protein levels (https://kmplot.com/analysis/). **G** *PILRB* mRNA levels were significantly upregulated in tumor tissues compared with paired normal tissues (*n* = 12). **H** Western blotting analysis demonstrated higher levels of PILRB protein in tumor tissues compared with paired normal tissues (*n* = 12). **I** Representative IHC images (Left) and IHC score (Right) of PILRB protein levels in tumors and paired normal tissues (*n* = 49). **J** Survival analysis of 49 patients with STAD in relation to different PILRB expression levels (*P* < 0.05).

Exploring the function of PILRB in GC progression, we observed that in TCGA database, *PILRB* mRNA levels were significantly upregulated in stomach adenocarcinoma (STAD) samples compared with that in normal tissues (Fig. 1A). Regarding prognosis the following were observed: patients with advanced clinical stages had distinctly higher *PILRB* mRNA levels than normal tissues (Fig. 1B), cases having higher tumor grade exhibited increased *PILRB* expression compared to adjacent tissues (Fig. 1C), and patients with advanced LNM showed higher *PILRB* expression than normal tissues (Fig. 1D). In TCGA database, there is no significant difference in *PILRB* mRNA levels in tumor tissues of GC patients with different clinical stages, tumor grades, and LNM. Moreover, patients with GC in the GSE62254 dataset showing *PILRB* downregulation were significantly associated with better overall survival (OS) and first progression (PF) (Fig. 1E, F). In our cohort, qRT-PCR and western blotting (WB) assays showed that PILRB was upregulated at both the mRNA and protein levels in GC patient samples (Fig. 1G, H). However, there is no significant difference in the PILRB protein levels of GC patients with LN metastasis compared with patients without LN metastasis (Fig. S1A). Furthermore, the IHC assay showed that PILRB protein levels were higher in cancer samples (Fig. 1I). We then performed survival analysis in our cohort and found that patients with high PILRB levels showed poor OS (Fig. 1J). These results demonstrate that *PILRB* is upregulated in GC and may be a potential prognostic indicator for patients with GC.

### PILRB expression accelerates GC cell growth

**Fig. 2:**
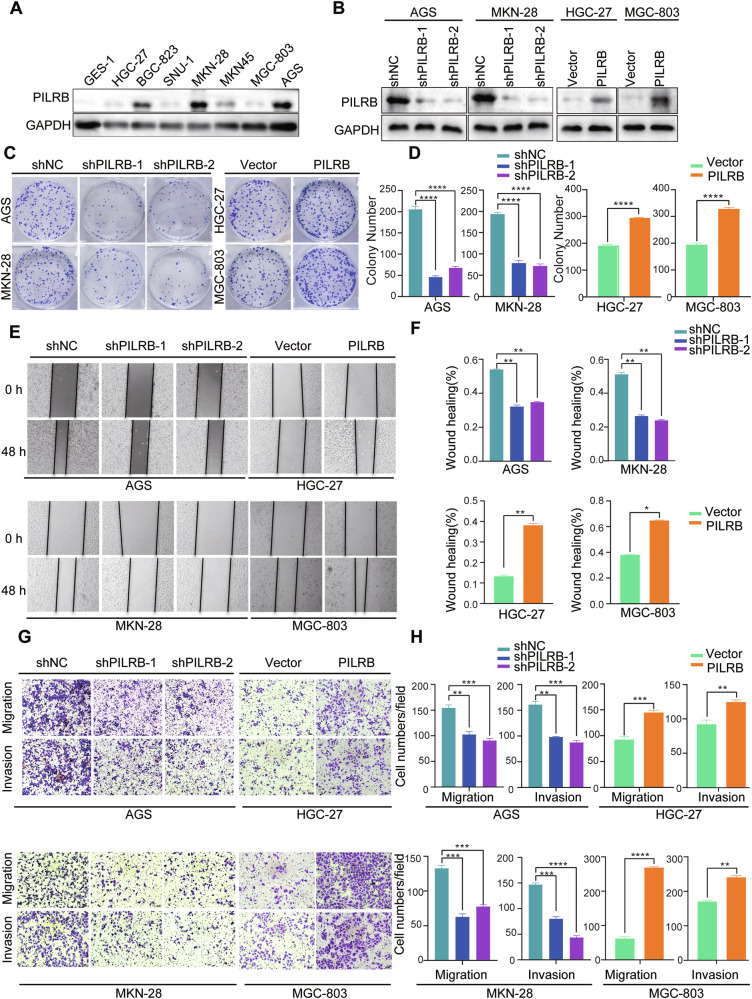
PILRB promotes proliferation and metastasis in GC cells in vitro. **A** Immunoblotting of PILRB expression in human HNSCC cells (GES-1, HGC-27, BGC-823, SNU-1, MKN-28, MKN45, MGC-803, AGS). **B** Western blotting of normal control (shNC) versus PILRB knockdown with sh#1, sh#2 in AGS and MKN-28, and vector versus PILRB overexpression (PILRB) efficiencies in HGC-27 and MGC-803 cells. **C**, **D** Cell colony formation assays of PILRB knockdown and PILRB overexpression GC cells were recorded (**C**) and quantitatively analyzed (**D**). **E**, **F** Wound-healing assay of PILRB knockdown and overexpression GC cells were recorded (**E**) and quantitatively analyzed (**F**). **G**, **H** Images, and quantification of transwell migration and invasion assays of PILRB knockdown and overexpression GC cells were recorded (**G**) and quantitatively analyzed (**H**).

To investigate the relationship between PILRB and GC progression, we measured PILRB protein levels in GC cells and selected four cell lines (AGS, MKN-28, HGC-27, and MGC-803) with different PILRB expression levels to perform further experiments (Fig. 2A). We knocked down *PILRB* and constructed stable *PILRB*-overexpressing GC cells, and then validated the efficacy of PILRB protein levels by WB (Fig. 2B). Further, in vitro loss- and gain-of-function experiments were conducted to explore the role of *PILRB* in the progression of GC. *PILRB* knockdown by shRNA in AGS and MKN-28 cells significantly inhibited their proliferation and clonogenicity. In contrast, ectopic expression of *PILRB* in HGC-27 and MGC-803 cells remarkably increased their proliferation and clonogenicity (Fig. 2C, D and Fig. S2A). These results indicated that *PILRB* plays an oncogenic role in GC.

### PILRB promotes GC cell migration and invasion

To evaluate the effects of PILRB on cell migration and invasion, it was observed that epithelial-to-mesenchymal transition (EMT) markers were upregulated by PILRB. The expression of mesenchymal markers (N-cadherin and vimentin) was suppressed, while the expression of epithelial markers (E-cadherin) was elevated in *PILRB* knockdown by shRNA in AGS and MKN-28 cells. In contrast, ectopic expression of *PILRB* in HGC-27 and MGC-803 cells remarkably increased the expression of mesenchymal markers (N-cadherin and vimentin) and inhibited the expression of epithelial markers (E-cadherin) (Fig. S2B). As determined by wound-healing and Matrigel invasion assays, knockdown of *PILRB* markedly inhibited the migration and invasion abilities of AGS and MKN-28 cells, whereas *PILRB* overexpression significantly increased the migration abilities of HGC-27 and MGC-803 cells (Fig. 2E–H). These findings demonstrated that *PILRB* plays an important role in promoting the migration and invasion of GC cells.

### PILRB activates PI3K/AKT signaling pathway

**Fig. 3:**
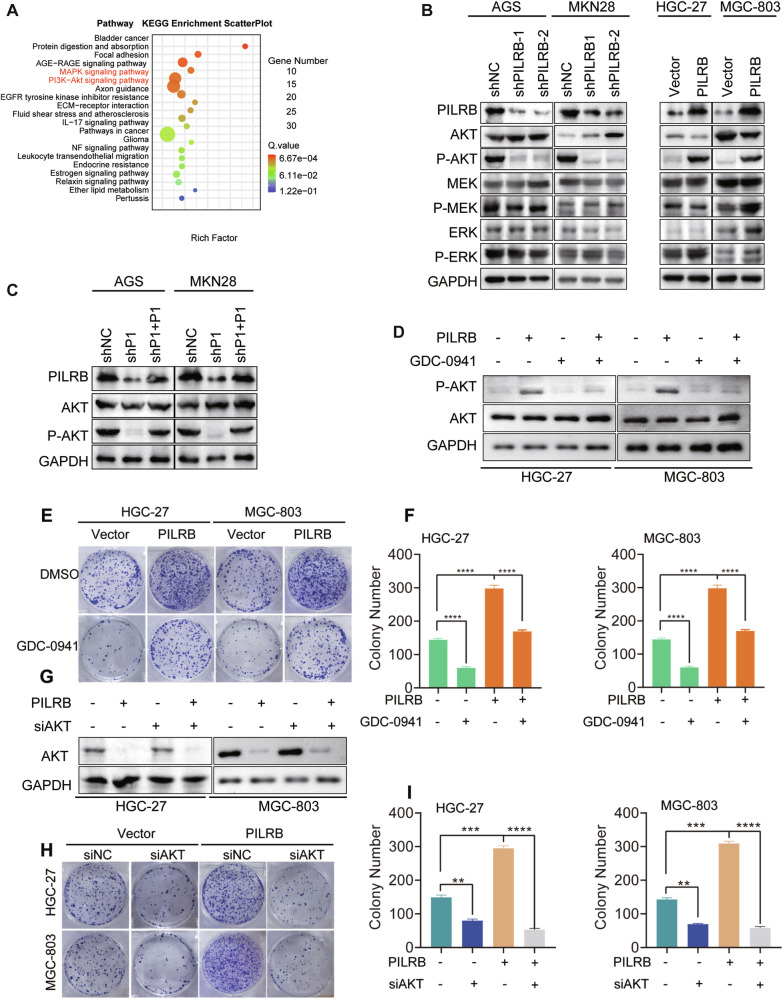
PILRB stimulates the PI3K/AKT signaling pathway in GC cells. **A** KEGG pathway enriched by differently expressed genes influenced by PILRB in AGS. **B**, **C** Western blotting analysis detected that *PILRB* knockdown remarkably inhibited AKT phosphorylation while not affecting MEK and ERK phosphorylation, and *PILRB* overexpression substantially promoted AKT phosphorylation while not affecting MEK and ERK phosphorylation (**B**). Wild-type PILRB reversed inhibition of AKT phosphorylation caused by PILRB depletion (**C**). **D** Western blotting analysis shows that PI3K/AKT inhibitor GDC-0941 treatment decreased AKT phosphorylation. **E**, **F** Cell colony formation assays showed that the proliferation ability of GC cells was significantly inhibited by GDC-0941 treatment in PILRB overexpression GC cells (**E**) Quantitative analysis (**F**). **G** Western blotting analysis showing siAKT treatment decreased AKT expression. **H**, **I** Cell colony formation assays showed that the proliferation ability of GC cells was significantly inhibited by siAKT treatment in PILRB overexpression GC cells (**H**) Quantitative analysis (**I**).

To explore the molecular mechanisms underlying the oncogenic role of PILRB, we performed RNA-sequencing in the deficiency of *PILRB* and control AGS/MKN28 cancer cells. Notably, the Kyoto Encyclopedia of Genes and Genomes (KEGG) pathway enrichment analysis showed that *PILRB* might activate the ERK/MAPK and/or PI3K/AKT signaling pathways in GC cells (Fig. 3A and Fig. S3A). Gene ontology (GO) enrichment analysis from the RNA-seq results demonstrated that differentially expressed genes (DEGs) of GC cells with or without *PILRB* knockdown were enriched in extracellular matrix organization, plasma membrane, and cell migration, which were associated with the process of EMT (Fig. S3B, C). Next, we performed a WB assay to examine the activation of the ERK/MAPK and PI3K/AKT signaling pathways and found that *PILRB* knockdown suppressed the activation of the PI3K/AKT pathway while not affecting the ERK/MAPK pathway. Meanwhile, *PILRB* overexpression stimulated the PI3K/AKT pathway while not influencing the ERK/MAPK pathway (Fig. 3B). Consistent with these results, the suppression of the PI3K/AKT pathway caused by *PILRB* depletion was reversible by overexpression of *PILRB* (Fig. 3C). To test whether the oncogenic function of *PILRB* was dependent on PI3K/AKT activation, *PILRB*-overexpressing GC cells were treated with GDC-0941 (a PI3K/AKT inhibitor), which inhibited AKT phosphorylation (Fig. 3D) and abolished the growth-promoting effect of *PILRB* expression, as determined by colony formation assays (Fig. 3E, F). In addition, siAKT abolished the proliferative ability of *PILRB-*overexpressing GC cells, demonstrating that PILRB promotes GC growth by stimulating the PI3K/AKT pathway (Fig. 3G–I). These results demonstrated that *PILRB* promotes GC progression by activating the PI3K/AKT pathway.

### PILRB interacts with and stabilizes IRS4 protein to stimulate PI3K/AKT pathway

**Fig. 4:**
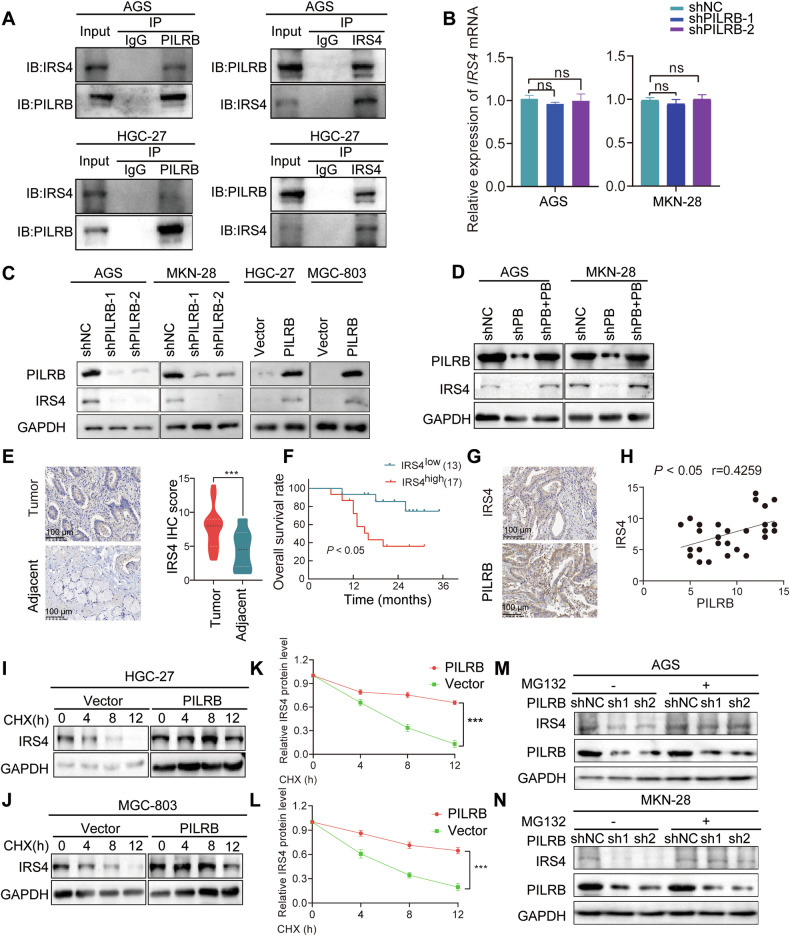
PILRB interacts with and stabilizes IRS4 to activate the PI3K/AKT signaling pathway in GC cells. **A** AGS and HGC-27 cells were treated with MG132 for 8 h and harvested for co-IP assay by western blotting. **B** The level of IRS4 mRNA expression in *PILRB* knockdown AGS and MKN-28 cells. **C**, **D** *PILRB* knockdown remarkably inhibited IRS4 protein levels, and *PILRB* overexpression substantially increased IRS4 protein levels (**C**). Wild-type PILRB reversed the changes in IRS protein levels caused by PILRB depletion (**D**). **E** Representative IHC images (Left) and IHC score (Right) of IRS4 protein levels in tumors and adjacent normal tissues. **F** Overall survival analysis of 30 GC patients showed that cases with higher IRS4 expression levels had a poorer prognosis (*P* < 0.05). **G**, **H** Representative images of IHC staining (**G**) and correlational analysis (**H**) between PILRB and IRS4 IHC score in our patients’ specimens. **I**–**L** IRS4 expression levels in GC cells treated with cycloheximide (CHX) for the indicated time points (**I, J**) and relative protein levels (**K**, **L**). **M**, **N** Western blotting analysis showing IRS4 expression levels after treatment with MG132 for 4 h in PILRB depletion GC cells.

To identify proteins that directly interact with PILRB in GC cells, we conducted a co-IP assay followed by LC/MS (Fig. S4A). PILRB-binding candidates were identified by comparing the anti-FLAG-PILRB IP products of FLAG-PILRB-overexpressing cells with those of the control AGS cells (Fig. S4B). Among the identified potential candidates, we focused on IRS4, which plays a central role in the signal transduction of cell surface receptors and the PI3K/AKT signaling pathway. The interaction of endogenous PILRB and IRS4 in AGS and HGC-27 cells by immunoprecipitation-Western blotting analyses was confirmed, demonstrating the interaction between PILRB and IRS4 (Fig. 4A). We then evaluated whether PILRB modulated the mRNA or protein levels of IRS4 by interacting with each other. *PILRB* depletion did not alter *IRS4* mRNA levels in GC cells (Fig. 4B). However, in *PILRB* knockdown AGS and MKN-28 cells, IRS4 protein levels were downregulated, whereas ectopic *PILRB* expression increased IRS4 protein levels (Fig. 4C). Rescue experiments also showed that IRS4 expression was reversed by overexpression of *PILRB* (Fig. 4D). These results demonstrated that *PILRB* increased IRS4 protein levels in GC cells.

To explore the relationship between PILRB and IRS4 expression levels in patients with GC, the IHC score showed that IRS4 was more highly expressed in GC tumor tissues than in normal tissues (Fig. 4E). Kaplan–Meier plots revealed that patients with high *IRS4* expression showed poor OS, similar to the results in the GEO and TCGA datasets (Fig. 4F and Fig. S5A). More importantly, IRS4 protein levels were positively correlated with PILRB protein levels in GC tissues (Fig. 4G, H). To explore the role of IRS4 in GC, we assessed the growth, migration, and invasion of *IRS4*-depleted and overexpressed GC cells. The results showed that IRS4 accelerated the proliferation (Fig. S5B) and enhanced the migration and invasion abilities of GC cells (Fig. S5C, D). These results indicate that IRS4 also plays an oncogenic role in patients with GC.

Further experiments showed that *IRS4* silencing significantly suppressed the PI3K/AKT signaling pathway in GC cells, whereas *IRS4* overexpression activated the PI3K/AKT signaling pathway (Fig. S6A). Consistent with the above results, overexpression of *IRS4* partly rescued the activation of the PI3K/AKT signaling pathway in *PILRB*-depleted GC cells (Fig. S6B), demonstrating that IRS4 is a crucial downstream target of PILRB that activates the PI3K/AKT signaling pathway in GC cells.

We next determined the underlying mechanisms by which PILRB upregulates IRS4 expression in GC cells. We found that PILRB maintained the stability of IRS4 in the presence of CHX (Fig. 4I–L). Next, we used the lysosome inhibitor bafilomycin A1 (Baf-A1) and the proteasome inhibitor MG132 and observed that IRS4 degradation was restored by MG132 in AGS cells treated with the protein synthesis inhibitor CHX (Fig. S6C). Analyzing the relationship between IRS4 protein stability and PILRB, we found that treatment with MG132 increased IRS4 expression levels in shNC/shPILRB-1/shPILRB-2 GC cells. Moreover, IRS4 protein levels triggered by proteasome inhibition could not be further decreased by *PILRB* depletion (Fig. 4M, N). Our data indicate that *PILRB* upregulates IRS4 expression through the proteasomal pathway.

### PILRB relieves IRS4 ubiquitination through deubiquitinase OTUB1

**Fig. 5:**
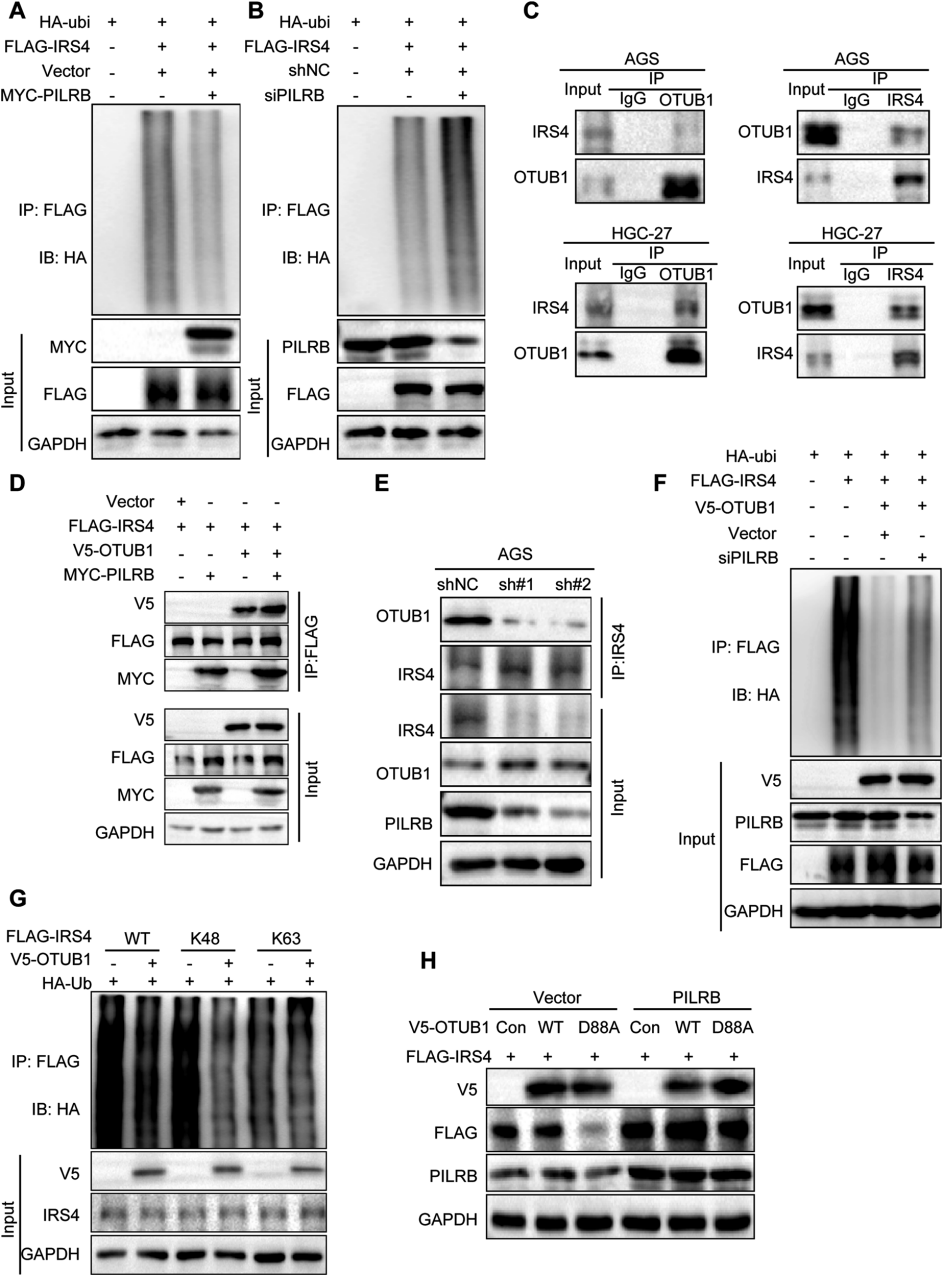
PILRB protects IRS4 from ubiquitin-regulated degradation by combining with OTUB1 in GC cells. **A** HEK293T cells were transfected with FLAG-IRS4, PILRB siRNA, and HA-Ub as indicated. Cells were harvested and lysed, immunoprecipitated by FLAG M2 beads, and subjected to immunoblotting with the HA antibody to detect IRS4 ubiquitination. **B** HEK293T cells were transfected with FLAG-IRS4, PILRB plasmid, and HA-Ub as indicated. Cells were harvested and lysed, immunoprecipitated by FLAG M2 beads, and subjected to immunoblotting with the HA antibody to detect IRS4 ubiquitination. **C** AGS and HGC-27 cells were treated with MG132 for 8 h and harvested for co-IP assay by western blotting. **D** HEK293T cells were transfected with FLAG-IRS4, MYC-PILRB, V5-OTUB1, and HA-Ub as indicated. Cells were harvested and lysed, immunoprecipitated by FLAG M2 beads, which showed that PILRB increased OTUB1 binding to IRS4. **E** Endogenous Co-IP of IRS4 in AGS-shNC/AGS-shPILRB-1/2. Immunoblotting assay showed that OTUB1 binding to IRS4 was reduced in PILRB depletion AGS cells. **F** HEK293T cells were transfected with FLAG-IRS4, siPILRB, V5-OTUB1, and HA-Ub as indicated. Cells were harvested and lysed, immunoprecipitated by FLAG M2 beads, and subjected to immunoblotting with the HA antibody to detect IRS4 ubiquitination. **G** Co-IP assay was performed to analyze the ubiquitination of IRS4 in HEK293T cell cotransfected with FLAG-IRS4 and HA-Ub or its lysine residue mutants HA-Ub K48 or K63 together with or without V5-OTUB1. **H** AGS-Vector/AGS-PILRB cells were transiently transfected with V5-OTUB-Con/WT/D88A and FLAG-IRS4 plasmids. Immunoblotting assay showed that the IRS4 protein in the WT group was more stable compared to the D88A group.

To study whether PILRB affects IRS4 ubiquitination, we performed ubiquitination assays in HEK293T cells with ectopic FLAG-IRS4 and HA-Ub in the presence of MYC-PILRB or siPILRB and found that PILRB decreased the ubiquitination of IRS4 (Fig. 5A, B). To identify the deubiquitinase (DUB) that interacts with IRS4, we used an online database (BioGRID, IntAct) and performed Venn analysis with all known DUBs, and found that OTUB1 and USP9X proteins may potentially interact with IRS4 (Fig. S7A). OTUB1 might bind to IRS4 using protein mass spectrometry [21]. To validate which deubiquitinase binds to IRS4, we transfected V5-OTUB1/USP9X and FLAG-IRS4 vectors into HEK293T cells and performed immunoprecipitation-western blotting, which showed that only OTUB1 could bind to IRS4 (Fig. S7B). We confirmed the interaction between endogenous OTUB1 and IRS4 in AGS and HGC-27 cells using immunoprecipitation-western blotting, demonstrating the interaction between PILRB and OTUB1 (Fig. 5C).

We also explored whether the interaction between OTUB1 and IRS4 was influenced by PILRB and found that OTUB1 bound to IRS4 was significantly increased by MYC-PILRB (Fig. 5D). To further validate this, we conducted endogenous immunoprecipitation-western blotting assays in AGS cells in which PILRB was stably knocked down. The results showed that OTUB1 binding to IRS4 decreased with PILRB deficiency (Fig. 5E). We then sought to explore whether PILRB regulated the deubiquitination of IRS4 by impacting IRS4 binding to OTUB1. Our results revealed that the knockdown of PILRB reversed OTUB1-mediated deubiquitination of IRS4 (Fig. 5F).

To determine the deubiquitination of IRS4 by OTUB1, FLAG-IRS4, and HA-Ub K48 or K63 mutants were co-transfected with or without V5-OTUB1 into HEK293T cells. As shown in the results (Fig. 5G), OTUB1 decreased the ubiquitination of IRS4 in the presence of the WT and the K48-Ub mutant, but not in the presence of the K63-Ub mutant, demonstrating that OTUB1 removes the K48-linked polyubiquitination chains on IRS4.

OTUB1 stabilizes target proteins by inhibiting E2-conjugating enzymes. The D88 site of OTUB1 has been shown to be important for its deubiquitinase activity of OTUB1. Mutating the D88 site to A (D88A) attenuates the deubiquitinase function of OTUB1. To explore whether the PILRB-stabilizing IRS4 protein relies on OTUB1 deubiquitinase function, we transfected the D88A mutant of OTUB1 into vector- or PILRB-stable GC cells and found that the IRS4 protein in the OTUB1-WT group was more stable. However, the IRS4 protein was remarkably reduced in the OTUB1-D88A group, and this difference was more significant after *PILRB* overexpression (Fig. 5H). These results demonstrate that OTUB1 exerts its deubiquitin function by inhibiting the E2-conjugating enzyme leading to the stabilization of IRS4 protein.

### The oncogenic role of PILRB largely dependent on IRS4

**Fig. 6:**
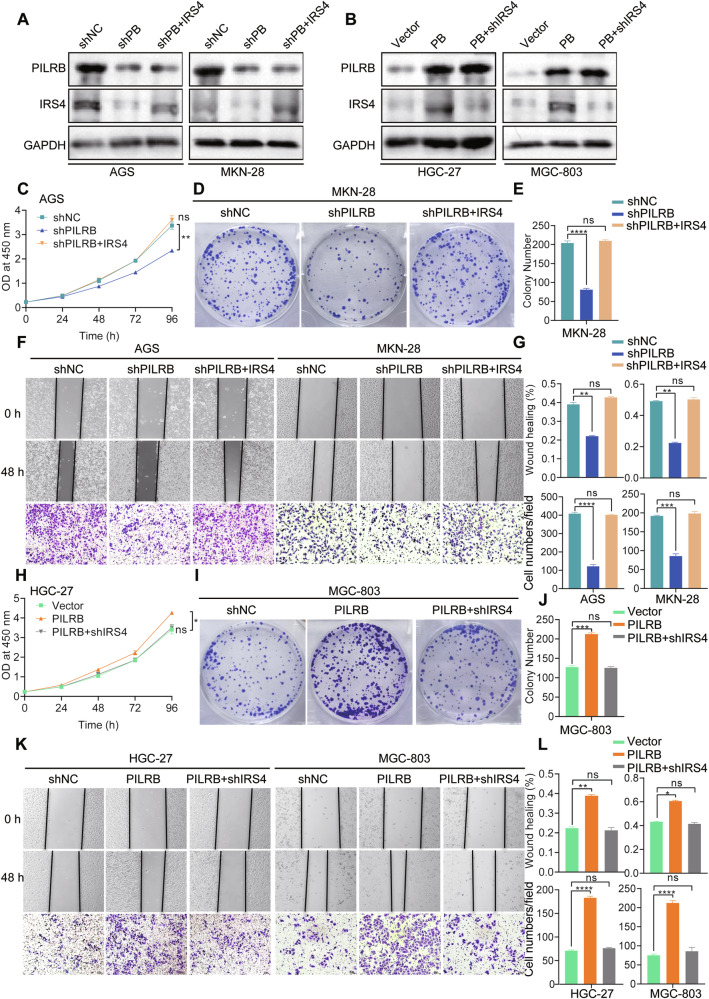
PILRB mediates IRS4 protein stability to promote proliferation and metastasis in GC cells in vitro. **A**, **B** WB analysis displaying IRS4 expression levels after IRS4 or shIRS4 transfer in PILRB-depleted or PILRB-overexpressed GC cells. **C** CCK8 assays were performed to explore the proliferation ability of AGS-shNC/AGS-shPILRB treated with vector and IRS4 as indicated. **D**, **E** Cell colony formation assays were performed in MKN-28-shNC/MKN-28-shPILRB treated with vector and IRS4 as indicated and quantitatively analyzed (**E**). **F**, **G** The ability of migration and invasion were performed in shNC/ shPILRB GC cells treated with vector and IRS4 as indicated (**F**), and quantitatively analyzed (**G**). **H** CCK8 assays were performed to explore the proliferation ability of HGC-27-vector/AGS-PILRB treated with shNC and shIRS4 as indicated. **I**, **J** Cell colony formation assays were performed in MGC-803-vector/ MGC-803-PILRB treated with shNC and shIRS4 as indicated (**I**), and quantitatively analyzed (**J**). **K, L** The ability of migration and invasion were performed in vector/ PILRB GC cells treated with shNC and shIRS4 as indicated (**K**), and quantitatively analyzed (**L**).

To investigate the effects of IRS4 on *PILRB*-regulated cell proliferation and metastasis, AGS and MKN-28 cells stably transfected with shPILRB or shNC were transfected with IRS4 overexpression, and HGC-27 and MGC-803 cells stably transfected with PILRB or vector were transfected with shIRS4. Western blotting was performed to validate the transfection efficiency of these GC cells (Fig. 6A, B). As expected, IRS4 overexpression reversed the suppression of proliferation (Fig. 6C), clonogenicity (Fig. 6D, E), migration, and invasion (Fig. 6F, G) of AGS and MKN-28 cells induced by *PILRB* depletion. Consistent with the above results, *IRS4* knockdown in GC cells remarkably abolished the promoting function of PILRB on the proliferation (Fig. 6H), clonogenicity (Fig. 6I, J), migration and invasion abilities of HGC-27 and MGC-803 cells (Fig. 6K, L). These findings confirm that the oncogenic effect of *PILRB* on GC cells is partially dependent on IRS4 expression.

### PILRB augments cholesterol biosynthesis

**Fig. 7:**
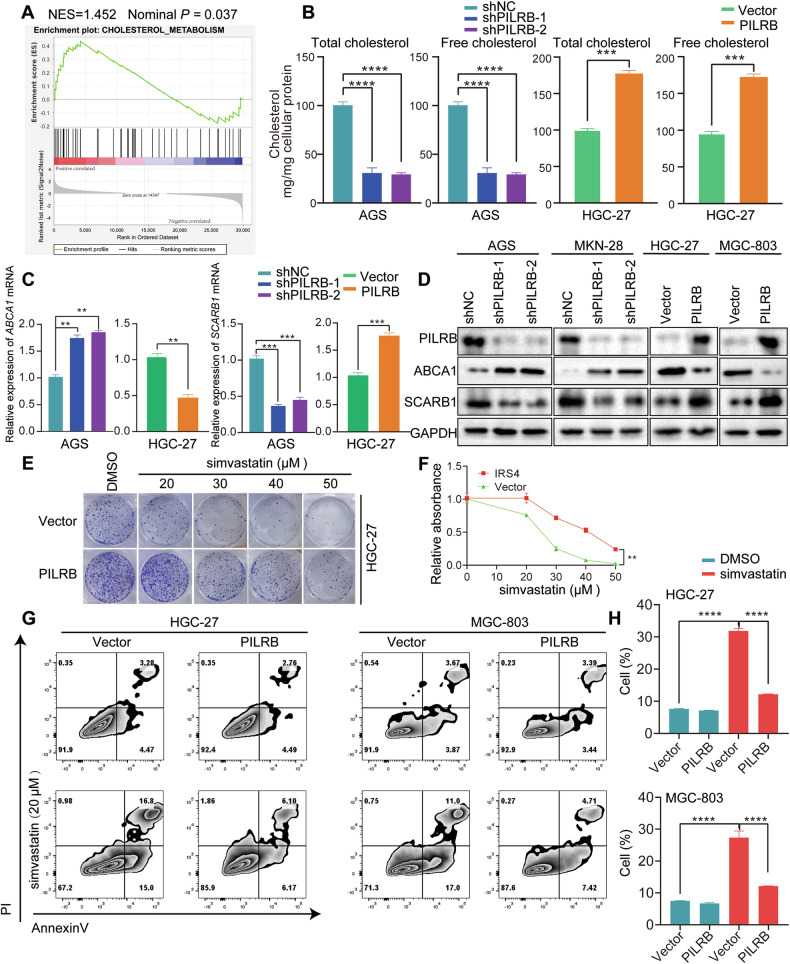
PILRB augments cholesterol biosynthesis in GC cells. **A** GSEA analysis of our transcriptome sequencing data enriched the cholesterol metabolism. **B** Levels of total cholesterol and free cholesterol in GC cells with PILRB depletion and overexpression. **C**, **D** RT-qPCR (**C**) and western blotting (**D**) analysis showing the ABCA1 and SCARB1 expression levels after PILRB depletion and overexpression of GC cells. **E**, **F** Cell colony formation assays showed that the proliferation ability of GC cells was significantly inhibited by simvastatin treatment in PILRB overexpression GC cells **(E)** and quantitatively analyzed (**F**). **G**, **H** PILRB overexpression GC cells were treated with simvastatin (20 μM), and apoptosis was analyzed with FACS (**G**) and quantitatively analyzed (**H**).

Interestingly, we also noticed that cholesterol metabolism was also enriched in our transcriptome sequencing data by GSEA analysis, possibly suggesting the stimulation of cholesterol biosynthesis by PILRB expression in GC cells (Fig. 7A). To further explore the mechanism underlying the role of PILRB in involving cholesterol metabolism, we firstly detected the levels of cholesterol in GC cells and observed a significant reduction in cholesterol synthesis (Fig. 7B). Furthermore, the expression of *ABCA1*, which can induce efflux of cholesterol, was increased, while *SCARB1*, which mediates selective uptake or influx of HDL-derived cholesteryl esters into cells and tissues was reduced after *PILRB* depletion in GC cells. Consistently, the expression of ABCA1 was reduced while *SCARB1* was elevated after PILRB overexpression in GC cells (Fig. 7C, D). These results proved that PILRB promote cholesterol biosynthesis through regulating the enzyme associated with cholesterol metabolism.

Since cholesterol is very important for sustaining membrane integrity and cell viability, a widely prescribed medication-simvastatin was used to control GC progression by inhibiting cholesterol levels. However, the results are still controversial, especially since there are no biomarkers to evaluate the efficacy of simvastatin therapy in GC patients. As we verified *PILRB* expression could increase cholesterol levels in GC cells, we investigated the involvement of *PILRB* regarding sensitivity to simvastatin. Ectopic expression of *PILRB* in GC cells clearly leads to resistance to simvastatin by colony formation assay (Fig. 7E, F). Furthermore, PILRB-overexpression GC cells led to a significant decrease in the percentage of apoptotic cells (Annexin-V/PI) caused by simvastatin (Fig. 7G, H). Together, these results confirmed PILRB-induced simvastatin resistance via elevating the cholesterol level in GC cells and indicate that *PILRB* may be a novel target for simvastatin treatment in GC patients.

### PILRB promotes tumorigenicity and metastasis in vivo

**Fig. 8:**
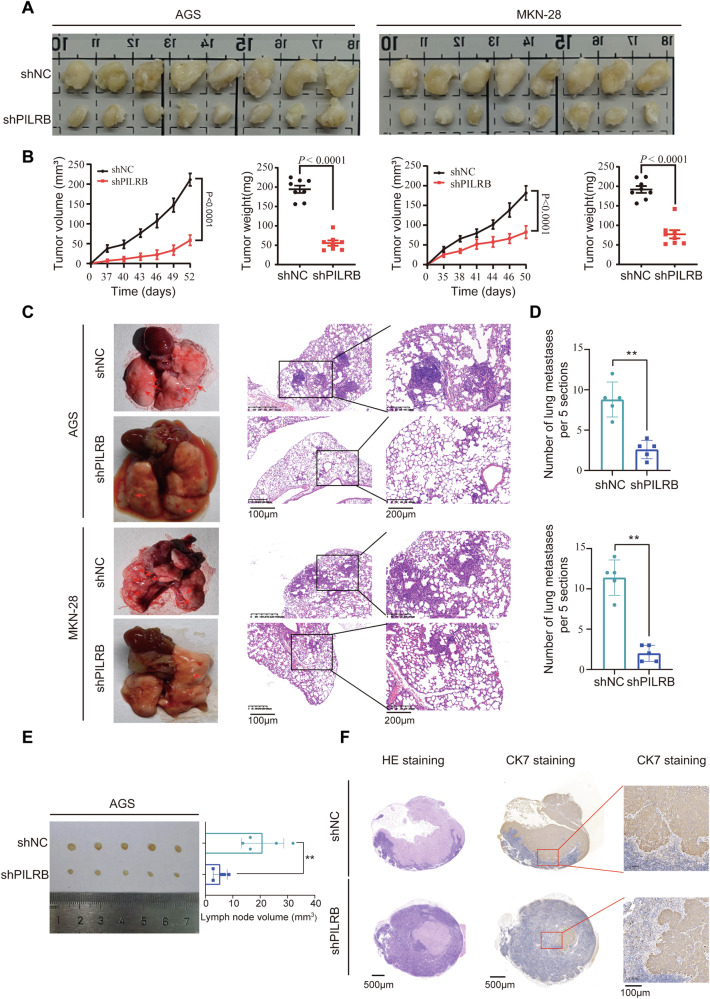
Effects of PILRB on GC cell growth and metastasis in vivo. **A**, **B** Photograph, and quantification of excised subcutaneous tumors obtained by injecting shNC versus shPILRB in two GC cell lines (*n* = 8 mice per group). **C**, **D** Representative specimen, and H&E staining images of the metastatic nodules of shNC versus shPILRB in two GC cell lines in the lung (**C**) and quantitatively analyzed (**D**). **E**, **F** Representative images of lymph nodes invaded by AGS-shNC/AGS-shPILRB cells with quantification of volume (**E**) and validated by CK7 IHC staining (**F**).

Based on the in vitro findings, we explored the effects of PILRB on GC promotion in vivo. The results showed that *PILRB*-depleted AGS/MKN-28 cells significantly suppressed the increase in tumor volume over the entire assay period and decreased the final tumor weight in the subcutaneous xenograft models (Fig. 8A, B). *PILRB* knockdown was verified by IHC staining of AGS xenografts. Cell proliferation was remarkably inhibited, as shown by Ki-67 staining, in *PILRB*-depleted xenografts compared with controls. IRS4 expression levels were also downregulated in *PILRB*-depleted cells (Fig. S8A–C).

We used nude mice to develop a lung metastasis model. Compared to mice in which the tail vein was injected with AGS and MKN-28-shPILRB cells, mice injected with AGS and MKN-28-shNC cells developed more lung metastatic nodes, confirmed through histologic examination (Fig. 8C, D). To further confirm the effects of PILRB on the lymphatic metastasis of GC, we used nude mice to establish a popliteal LN metastasis model. *PILRB*-depleted cells were injected into the footpads of nude mice. As expected, we found that *PILRB* depletion in GC cells resulted in smaller popliteal lymph nodes than in the shNC group (Fig. 8E). As cytokeratin 7 (CK7) is a specific marker for cancer cells, we examined the metastatic ratio via CK7. The results showed that the *PILRB* depletion group showed a remarkably lower lymphatic metastatic ratio (Fig. 8F). Taken together, the xenograft mouse models verified that PILRB promotes tumorigenesis and metastasis in GC cells.

## Discussion

PILRB was previously shown to be expressed mainly in NK cells, dendritic cells, and macrophages and is associated with the regulation of the biological functions of immune cells. However, to the best of our knowledge, its expression and functional roles in cancer cells have never been evaluated. In our report, we verified that PILRB was upregulated in gastric tumors compared to adjacent normal tissues, and its upregulation predicts poor survival outcomes. However, we observed no significant difference in the expression of PILRB protein in the tumor tissues of GC patients with and without metastasis by western blot (Fig. S1A), which might be caused by the limited number of GC patients with metastasis. In the future, a larger clinical sample cohort size would be valuable to verify the expression of the PILRB protein of GC patients in the tumor tissues of GC patients with and without metastasis Therefore, we investigated the oncogenic role of *PILRB* in GC cells both in vitro and in vivo. Depletion of *PILRB* in AGS and MKN-28 cells significantly inhibited cell proliferation and colony formation. Conversely, *PILRB* overexpression GC cells exhibited the opposite effects. Metastasis is a multistep process in which tumor cells undergo EMT, break away from their primary site, travel through the lymph or blood system, and arrive in and colonize new parts of the body. To build secondary tumors in tissue sites that lie some distance from the primary tumor, motile cancer cells must enter the blood vessels or lymphatic systems. After the invasion, it is very important for dissemination that motile cancer cells settle in adjacent lymph nodes instead of immediately moving to a distant tissue site. Lymph node metastasis is thought to be closely associated with the ability of cancer cells to build secondary tumors and is widely recognized as a key criterion in determining the cancer stage. We found overexpression of *PILRB* in HGC-27 and MGC-803 cells significantly enhanced their migratory and invasive abilities. Conversely, *PILRB* deficiency inhibited the ability of AGS and MKN-28 cell migration and invasion. Consistent with the in vitro results, lymph node metastasis, and tail vein metastasis models were established to validate the oncogenic role of PILRB in GC cells by injecting *PILRB*-deficient GC cells. Collectively, we verified that PILRB knockdown inhibited GC cell growth and metastatic potential in vitro and in vivo.

To further examine the molecular mechanism of PILRB-mediated oncogenic factors in GC, we identified the PI3K/AKT pathway as the major downstream signaling pathway underlying the oncogenic function of PILRB in GC cells. The PI3K/AKT pathway is known to play a critical role in cancer progression. Dysfunction of this pathway is associated with various diseases, including malignant tumors [22]. Previous studies reported that patients with GC show abnormal activation of the PI3K/AKT pathway, which may provide potential therapeutic targets for GC [23]. However, the mechanisms underlying the hyperactivation of the PI3K/AKT pathway in GC remains largely unknown. In our study, we elucidated that aberrant expression of *PILRB* in GC cells could induce abnormal activation of the PI3K/AKT pathway by stabilizing IRS4 protein. IRS4 is a member of the IRS family consisting of four closely related members, IRS1-IRS4, and two distant relatives, IRS5/DOK4 and IRS6/DOK5. IRS4 was first identified and characterized in the HEK293T human embryonic kidney cell line, where it responds to insulin [24]. However, in breast cancer cells, IRS4 can induce constitutive PI3K/AKT hyperactivation even in the absence of insulin or growth factors [25]. As a constitutively active gene, the expression level of IRS4 is strictly regulated by the transcriptional and translational levels in cells. Moloney murine leukemia virus (MuLV)-induced and mouse mammary tumor virus (MMTV)-induced insertional mutagenesis screens always target IRS4, leading to the upregulation of *IRS4* mRNA expression levels, underscoring the importance of IRS4 in tumorigenesis [25]. In T-cell acute lymphoblastic leukemia and subungual exostosis, chromosomal translocation events promote the activation of IRS4 mRNA transcription [26, 27]. Hsa _circ_0023409 enhances IRS4 expression, promoting GC proliferation and metastasis [28]. IRS4 expression levels were also much higher in HEK293T cells expressing T antigen-like 293T cells and adeno-associated virus (293AAV) than in the parental HEK293T cell line [29]. However, it remains unclear whether IRS4 protein levels are controlled at the translational level. Recently, a study showed that casein kinase 1gamma2 (CK1gamma2) can phosphorylate Ser389 of IRS4, resulting in promoting IRS4 degradation via CHIP through the ubiquitin/lysosome pathway [30]. In the present study, we first performed co-IP of PILRB, followed by protein sequencing for the identification of PILRB functional partners, which identified IRS4 as a potential binding partner. Co-IP assay further confirmed the interaction between PILRB and IRS4. IRS4 was found to be downregulated following deficiency of *PILRB*, meanwhile IRS4 levels were upregulated by *PILRB* overexpression in GC cells. Taken together, we illustrated that PILRB hyperactivates the PI3K/AKT pathway by stabilizing the IRS4 protein.

Next, we explored the molecular mechanisms through which PILRB stabilizes IRS4 in GC cells. Our results showed that *IRS4* mRNA was not altered when *PILRB* was knocked down or overexpressed, while IRS4 protein levels were elevated in *PILRB* overexpression in GC cells, suggesting that PILRB could stabilize IRS4 protein levels via post-translational modification. Cellular protein degradation is primarily caused by autophagy or the ubiquitin-proteasome (UPS) system. To identify the pathway through which PILRB regulates IRS4 protein levels, we treated GC cells with CHX, a protein synthesis inhibitor. In PILRB-overexpressing GC cells, we found that the degradation of IRS4 was markedly reduced compared to that in the vector group, demonstrating that PILRB regulates IRS4 protein levels depending on the stability of the UPS system. Furthermore, MG132 significantly reduced the ubiquitination of IRS4 in PILRB-overexpressing GC cells, revealing that PILRB stabilizes the IRS4 protein by reducing the ubiquitination of IRS4. We further used two online tools to predict the deubiquitinase DUB that interacts and stabilizes with IRS4. The results showed that OTUB1 might potentially interact with IRS4. Next, we demonstrated that PILRB relieves the ubiquitination of IRS4 through OTUB1, thereby increasing IRS4 protein expression.

Ovarian tumor domain-containing ubiquitin aldehyde binding protein 1 (OTUB1) is a non-canonical DUB that regulates specific ubiquitin-conjugating enzymes (E2_S_), which is involved in the malignant progression of different types of tumors [31]. As a tumor suppressor, OTUB1 suppresses cell proliferation and triggers p53-dependent apoptosis by regulating the DNA damage response [32]. However, OTUB1 can facilitate the development of esophageal squamous cell carcinoma by stabilizing the SNAIL protein [33]. OTUB1 also plays a crucial role in checkpoints during T cell-mediated antitumor immune responses [34]. To the best of our knowledge, this study represents the first investigation demonstrating that PILRB can recruit OTUB1 to stabilize IRS4 protein, thereby promoting GC progression.

Some studies have showed the anti-tumor effect of GC cells treatment of in virto, however, clinical trials in GC patients have no positive comes based on those preclinical results [35], which highlights the urgent need for novel predictive biomarkers of the efficacy of statins to treat GC. In our study, we found that PILRB could increase the cellular cholesterol level by altering the expression levels of ABCA1 and SCARB1 to induce resistance to statin therapy. All results demonstrate that clinical trials did not preselect GC patients with high PILRB levels may have limited the positive outcome. Hence, clinical trials with selective enrollment are suggested to consider the expression level of PILRB+ tumors. Previous studies showed that the PI3K/AKT pathway activated cholesterol metabolism through posttranslational regulation to regulate the expression level of ABCA1 and SCARB1 in the cell surface of HepG2 [36]. However, our results show that PILRB could regulate the expression level of ABCA1 and SCARB1 in GC cells, which suggests that other molecular mechanisms might regulate the expression level of ABCA1 and SCARB1 in GC cells independent of IRS4-mediated stimulation of the PI3K/AKT signaling pathway.

Overall, this study unveils that PILRB acts as an oncogenic protein to interact with IRS4 and recruit OTUB1 to deubiquitinate and stabilize IRS4, leading to PI3K/AKT activation to enhance GC progression. Our work also provides evidence that PILRB may prove to be of wider clinical relevance as a biomarker for PI3K/AKT pathway-dependent GC and other cancers. Moreover, PILRB might be a wider clinical biomarker for lowering cholesterol level-dependent gastric and other cancer types.

## Conclusion

The oncogenic role of PILRB is proved in gastric tumorigenesis, providing new insights into the regulation of PI3K/AKT signaling in GC and establishing *PILRB* as a biomarker for simvastatin therapy resistance in GC.

### Data sharing

Any reasonable requests for access to available data underlying the results reported in this article will be considered. Such proposals should be submitted to the corresponding author.

### Supplementary Information

See Table S1–S2, Figures S1–S8 in the Supplementary Material for comprehensive image analysis.

## Supplementary information

supplementary information

Raw western blot data
