# Supplementary material for: PILRB potentiates the PI3K/AKT signaling pathway and reprograms cholesterol metabolism to drive gastric tumorigenesis and metastasis
Source: Cell Death Dis. 2024 Sep 3;15(9):642. doi: 10.1038/s41419-024-07026-5 (PMC11372125; doi:10.1038/s41419-024-07026-5)
Supplement: Supplementary file 1 — supplementary information [file 41419_2024_7026_MOESM1_ESM.pdf]

SUPPLEMENTARY INFORMATION

PILRB potentiates PI3K/AKT signaling pathway and reprograms cholesterol metabolism to drive gastric tumorigenesis and metastasis

Xing Wang[#1], Yuanyuan Liu[#3], Qiuyan Zhao[#2], Xin Wang[#4], Yixin Chen[5], Li Hou[5], Danshao Tian[5], Zi-Mei Peng[6], Xiao-Jian Han[7], Tao Wang[7], Zhen Zhang[6], Fang-Fang Tou[8], Shan Huang[*8], Jun Rao[*9], Lixiao Chen[*3], Zhi Zheng[*8]

[*]Corresponding author: zhengxia_2007@163.com, Chenlixiao1201@163.com, raojun1986@126.com, huangshan91@aliyun.com.

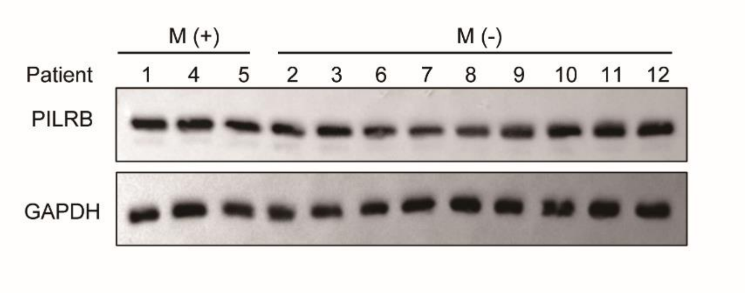

Fig. S1. The expression of PILRB in GC patients with or without metastasis: Western blot analysis of PILRB expression in 12 GC patients with or without metastasis. M (+): metastasis, M (−) non-metastasis.

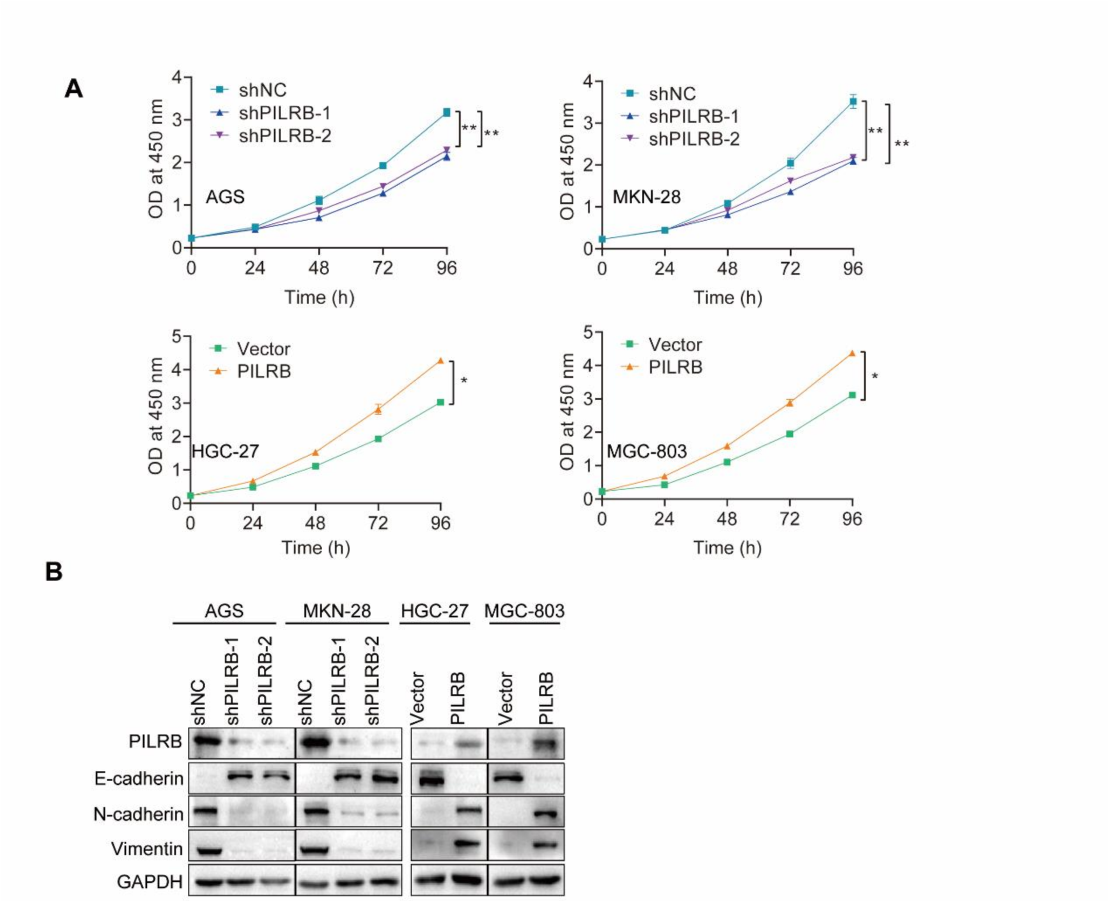

Fig. S2. PILRB promotes proliferation and metastasis in GC cells *in vitro.* A CCK8 assay showed that the proliferation ability of cells was significantly enhanced in PILRB-overexpressed and decreased obviously in *PILRB*-deficient GC cells. **B** Immunoblotting of EMT markers in GC cells after *PILRB* knockdown and overexpression.

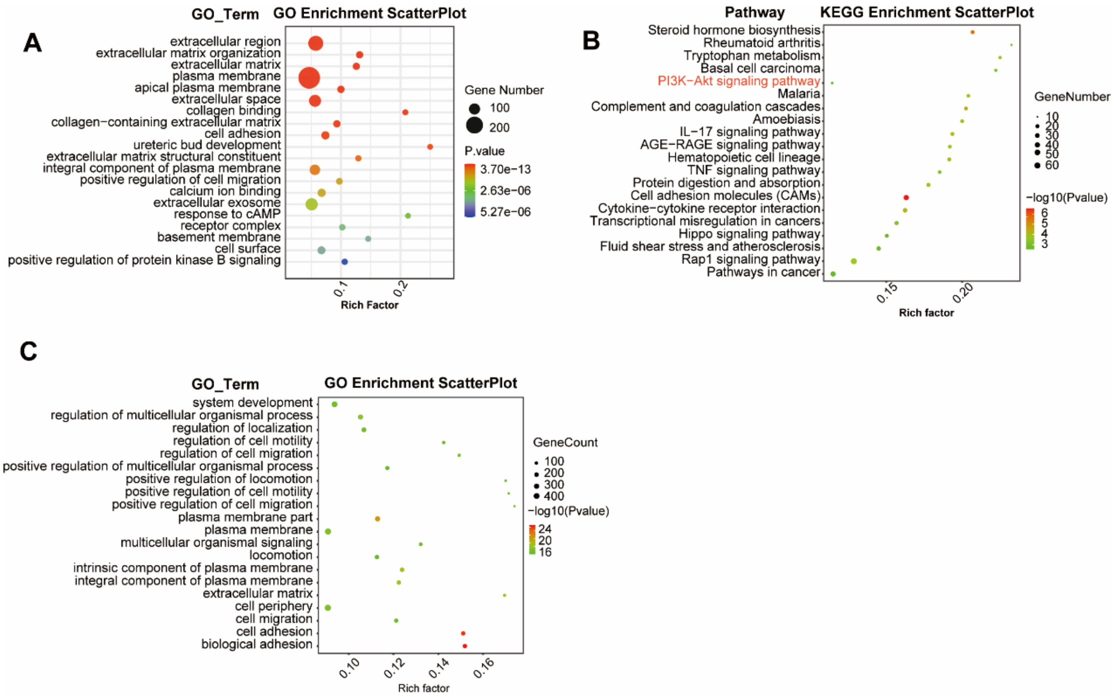

Fig. S3. KEGG pathways and GO analysis enriched by differently expressed genes influenced by PILRB in GC cells. A KEGG pathway enrichment analysis displaying the top 20 enriched pathways of DEGs in MKN-28 cells with or without *PILRB* knockdown. **B, C** GO analysis showing the top 20 enriched terms of biological process of DEGs in AGS (**B**) and MKN-28 (**C**) cells with or without *PILRB* knockdown.

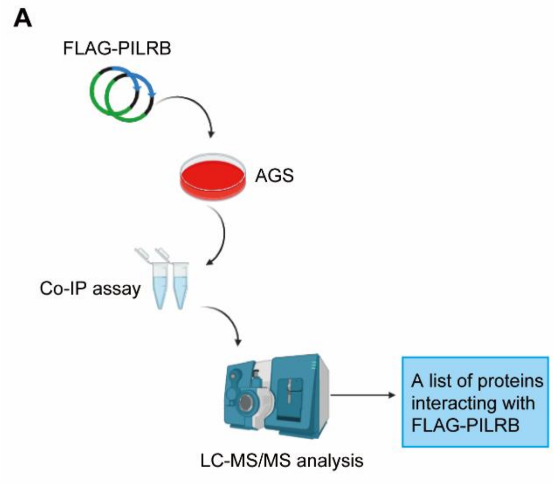

B

PILRB-associated proteins

Protein	Unique Peptides
PILRB	55
PILRA	28
IRS4	5

Fig. S4. Schematic of PILRB interactor discovery. A Liquid chromatography-mass spectrometry (LC-MS) identified IRS4 as a PILRB-binding protein. **B** The peptide numbers of IRS4 and PILRA associated with FLAG-PILRB.

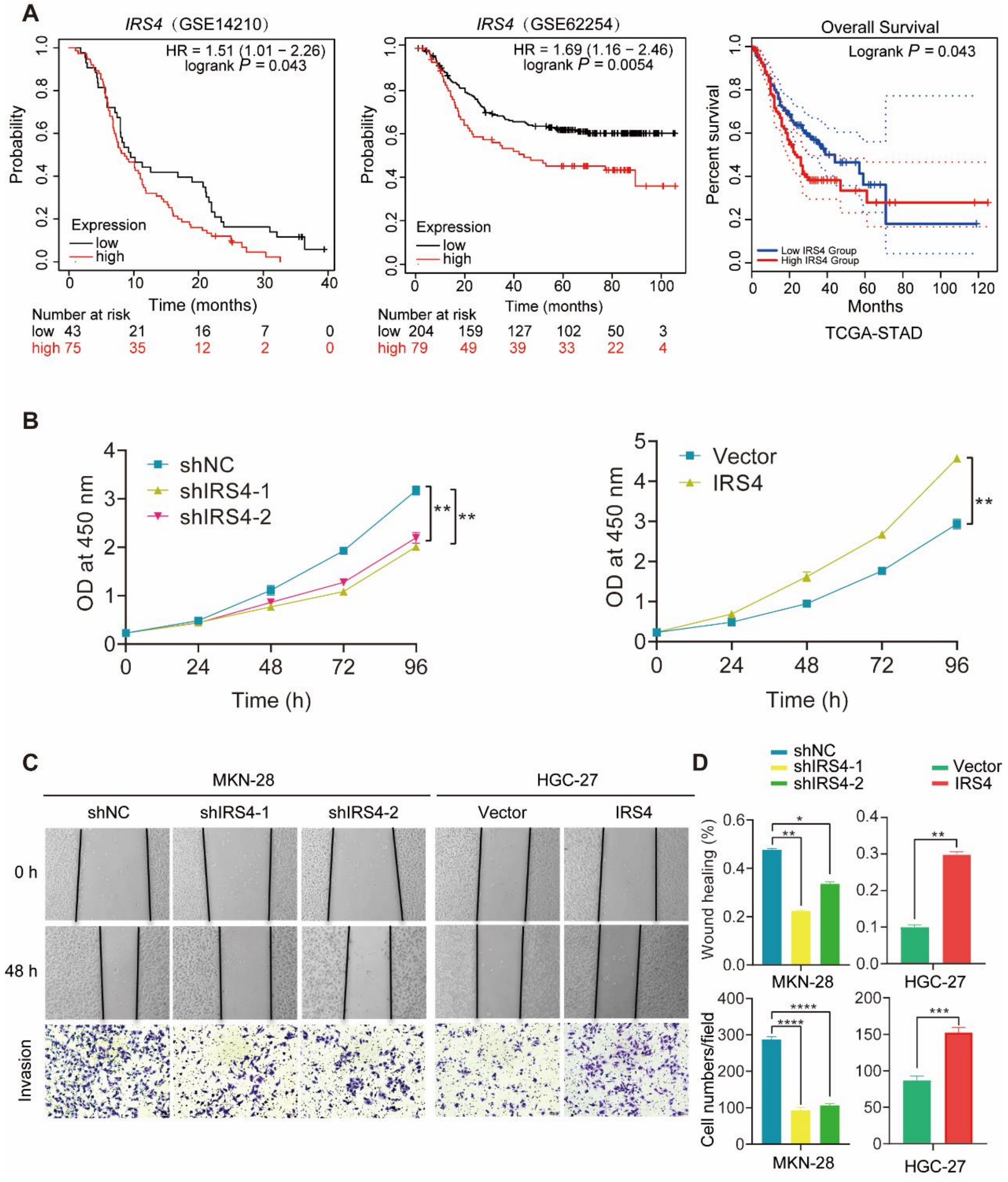

Fig. S5. PILRB promotes proliferation and metastasis in GC cells *in vitro*. A Two GEO datasets (GSE1420 and GSE62254) and TCGA dataset showed a significant association between elevated expression of *IRS4* and poor patient survival. **B** CCK8 assays were performed to explore the proliferation ability of *IRS4* knockdown and IRS4 overexpression GC cells. **C, D** Images and quantification of migration and invasion assays of *IRS4* knockdown and overexpression GC cells were recorded (**C**) and quantitatively analyzed (**D**).

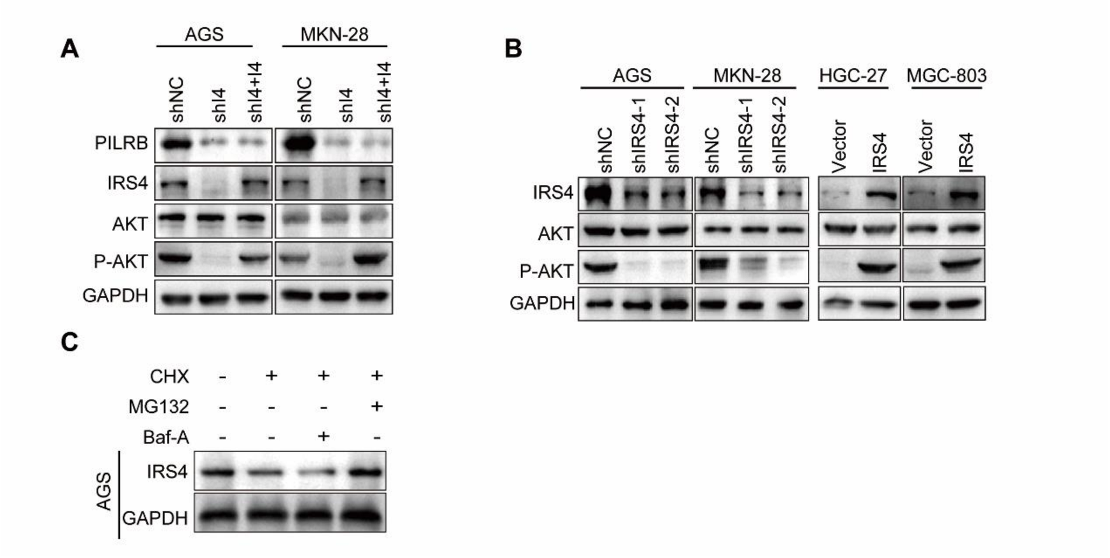

Fig. S6 PILRB stabilizes IRS4 to activate PI3K/AKT signaling pathway in GC cells. A, B Western blotting analysis detected that *IRS4* knockdown remarkably inhibited AKT phosphorylation, *IRS4* overexpression substantially promoted AKT phosphorylation (**A**), while *IRS4* rescued the inhibition of AKT phosphorylation caused by PILRB depletion (**B**). **C** Western blotting detection of IRS4 expression in AGS cells treated with MG132 or bafilomycin A1 and subjected to cycloheximide exposure.

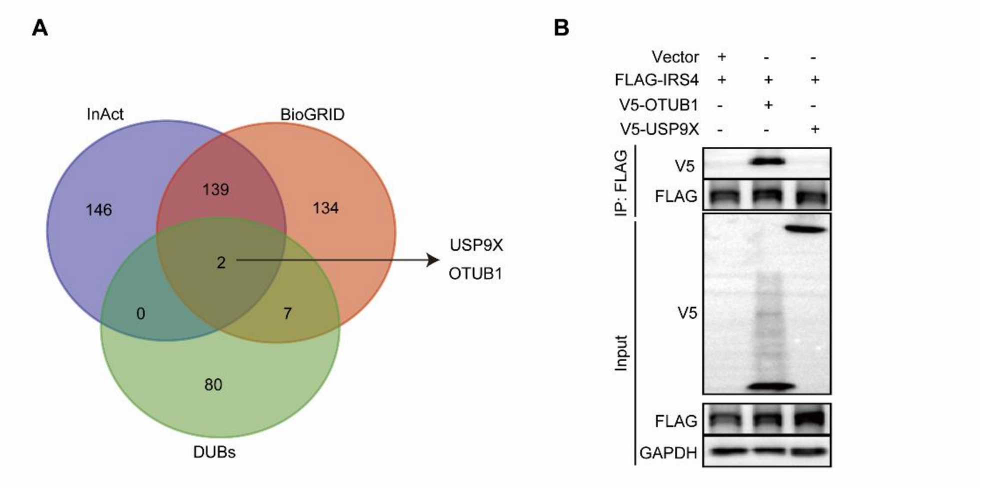

Fig. S7. OTUB1 binds to IRS4. A Predicting the DUBs that bind to IRS4 using public databases (BioGRID, IntAct) and Venn analysis with 73 known DUBs. Interaction proteins includes USP9X and OTUB1. **B** Co-IP experiment was performed to detect that only OTUB1 interacts with IRS4 but not USP9X in HEK293T cells.

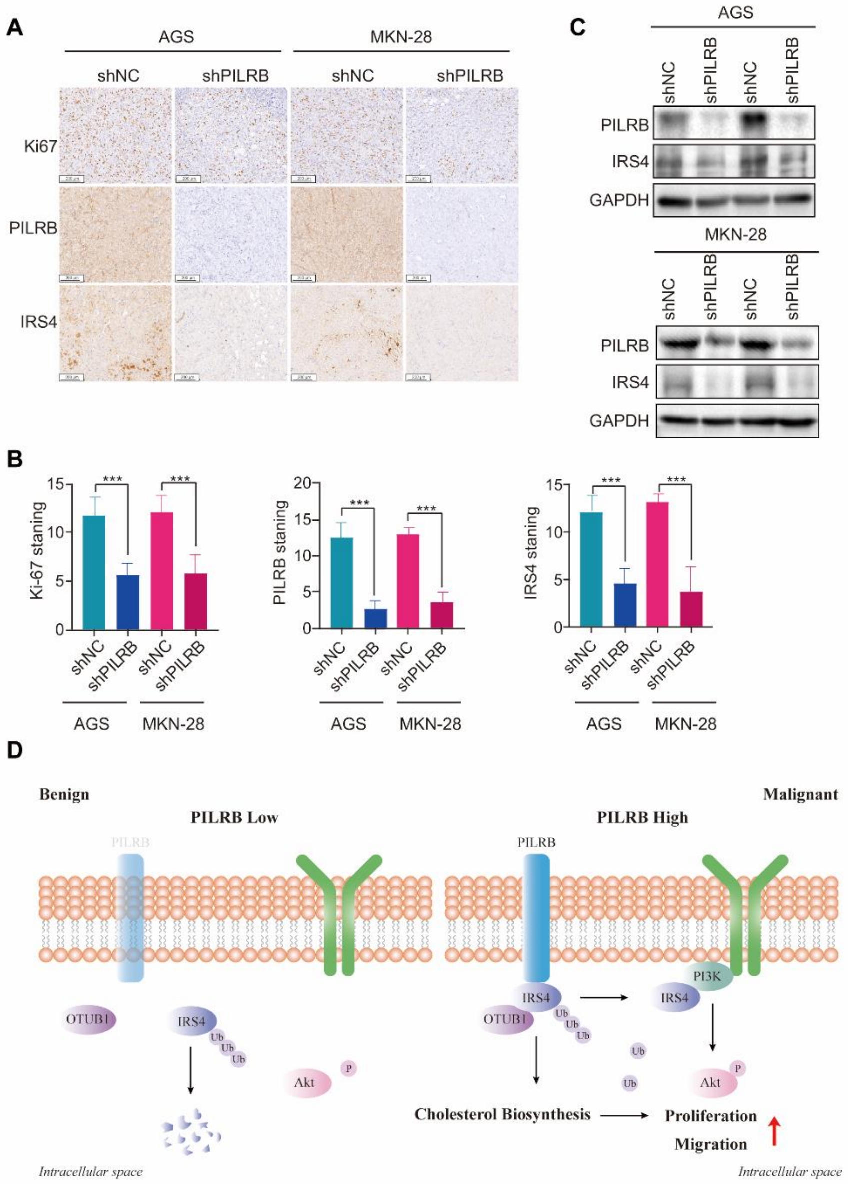

Fig. S8. Effects of PILRB on GC cells growth and metastasis *in vivo*. A, B IHC staining confirmed *PILRB* depletion in AGS and HGC-27 subcutaneous xenografts, which suppressed cell proliferation by Ki-67 level, and verified *PILRB* knockdown downregulated IRS4 protein level. **C** Western blot analysis further showed *PILRB* depletion in AGS and HGC-27 xenografts inhibited the IRS4 protein levels. **D** Graphical abstract describes how PILRB upregulates IRS4 levels and mediates overactivation of PI3K/AKT pathway to promote tumorigenesis and metastasis in GC cells.

Table S1: List of primary antibodies and their sources

Antibodies	SOURCE	IDENTIFIER
PILRB for WB	Invitrogen	PA5-42955
PILRB for IHC	Novus Biologicals	NBP2-98648
IRS4 for WB	Santa Cruz Biotechnology	sc-373778
IRS4 for IHC	Santa Cruz Biotechnology	sc-373778
OTUB1	abclonal	A11656
E-cadherin	proteintech	20874-1-AP
N-cadherin	proteintech	22018-1-AP
Vimentin	proteintech	10366-1-AP
FLAG	Absin	abs160020
GAPDH	proteintech	60004-1-Ig
MYC	Absin	abs149605
V5	Absin	abs149615
HA	proteintech	51064-2-AP
MEK1/2	Abcam	ab178876
phospho MEK1/2	Abcam	ab194754
ERK1/2	Abcam	ab17942
phospho ERK1/2	Abcam	ab76299
AKT	proteintech	10176-2-AP
phospho AKT	proteintech	80455-1-RR
ABCA1	proteintech	26564-1-AP
SCARB1	proteintech	21277-1-AP

Table S2: shRNA and main primer sequences

Oligonucleotides		
shPILRB-1: CCGGCGCCTTCCATTCACAAGGATTCTCGAG AATCCTTGTGAATGGAAGGCGTTTTTG	Sangon Biotech	N/A
shPILRB-2: CCGGCCATAGTTCCCAACGTGAGAACTCGAG TTCTCACGTTGGGAACTATGGTTTTTG	Sangon Biotech	N/A
shIRS4-1: CCGGCCATTCGCTATGATGCTGAAACTCGAG TTTCAGCATCATAGCGAATGGTTTTTG	Sangon Biotech	N/A
shIRS4-2: CCGGGCTGGTTTCAACCTGTTGCTACTCGAG TAGCAACAGGTTGAAACCAGCTTTTTG	Sangon Biotech	N/A
PILRB-F: AGAATCATGGCACCTAAGTCTGG	Sangon Biotech	N/A
PILRB-R: GACAGCCAATGCAACCCTG	Sangon Biotech	N/A
IRS4-F: CGACCAAGCGACAAGAAGACT	Sangon Biotech	N/A

IRS4-R: GGTTCCCGAGGAAAGAAGCG	Sangon Biotech	N/A
GAPDH-F: ACAACTTTGGTATCGTGGAAGG	Sangon Biotech	N/A
GAPDH-R: GCCATCACGCCACAGTTTC	Sangon Biotech	N/A
ABCA1-F: ACCCACCCTATGAACAACATGA	Sangon Biotech	N/A
ABCA1-R: GAGTCGGGTAACGGAAACAGG	Sangon Biotech	N/A
SCARB1-F: AATAAGCCCATGACCCTGAAGC	Sangon Biotech	N/A
SCARB1-R: GCCCCACATGATCTCACCC	Sangon Biotech	N/A
