# Supplementary material for: PILRB potentiates the PI3K/AKT signaling pathway and reprograms cholesterol metabolism to drive gastric tumorigenesis and metastasis
Source: Cell Death Dis. 2024 Sep 3;15(9):642. doi: 10.1038/s41419-024-07026-5 (PMC11372125; doi:10.1038/s41419-024-07026-5)

Figure 1H

PILRB

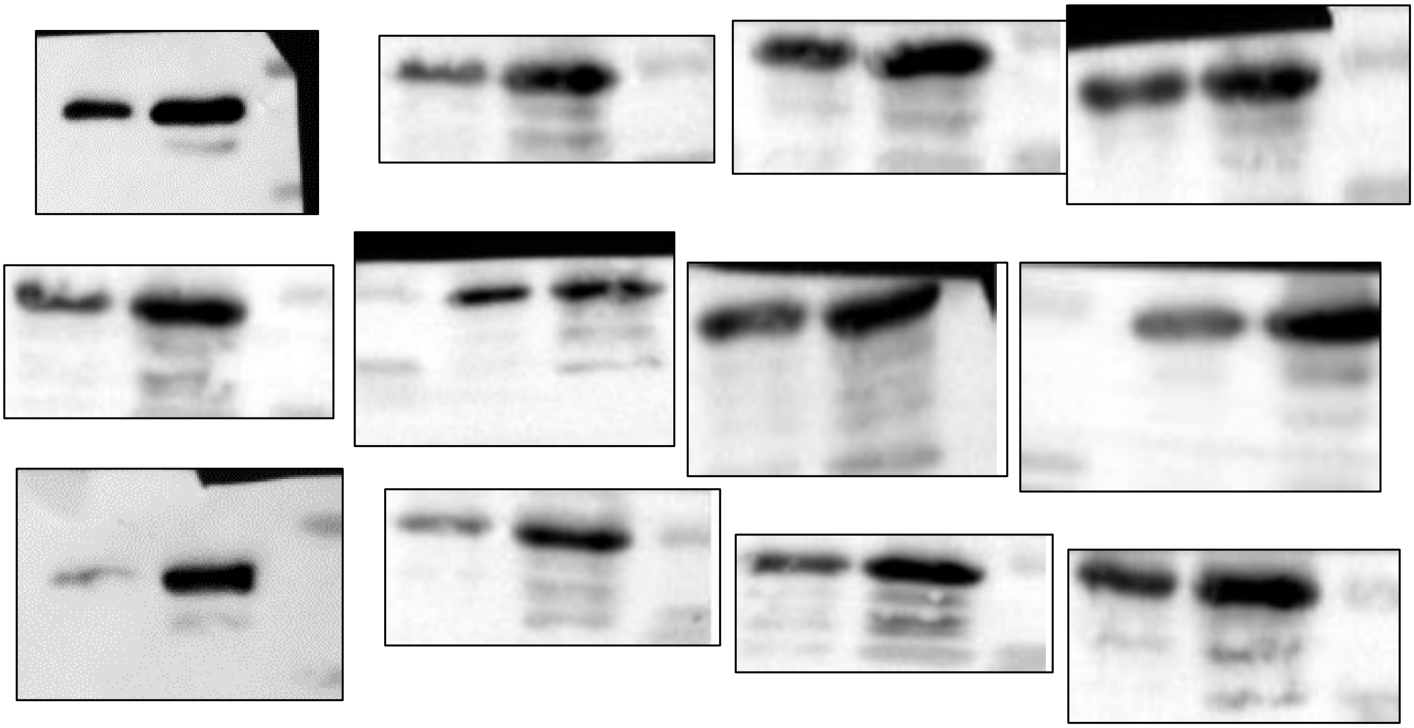

GADPH

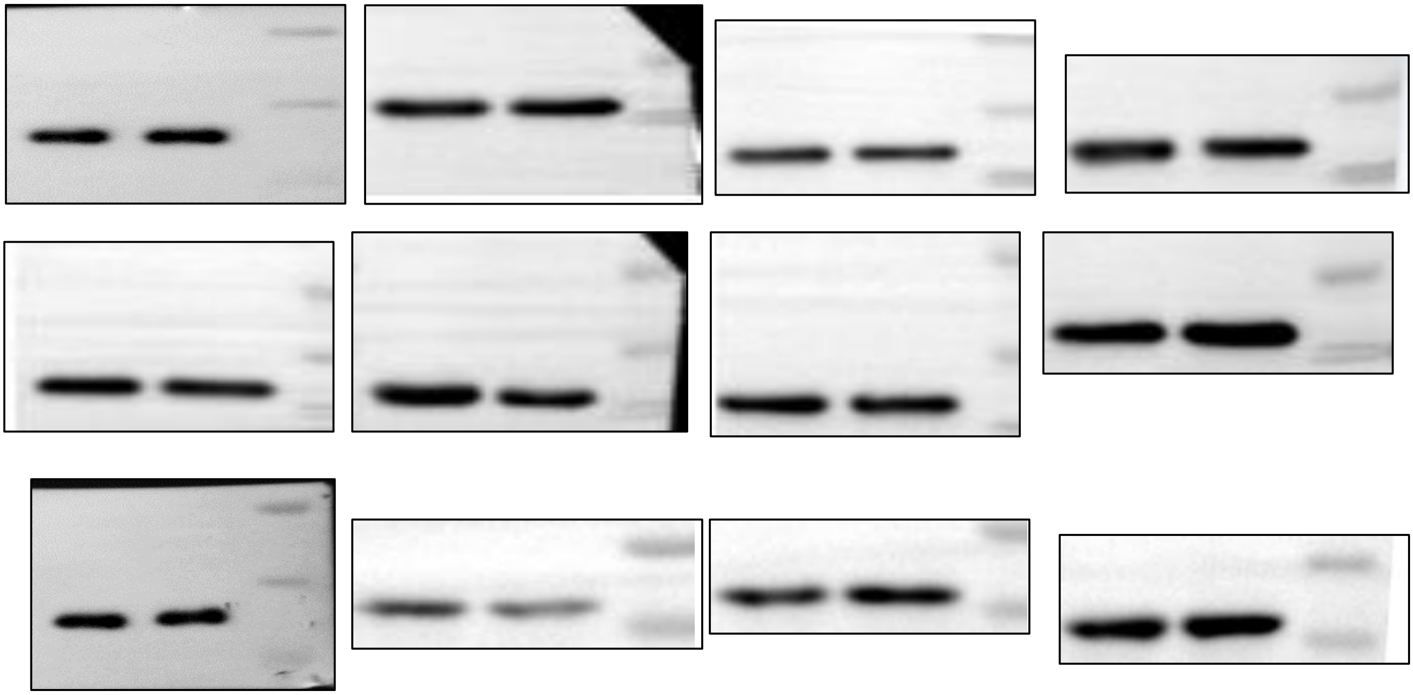

Figure 2A

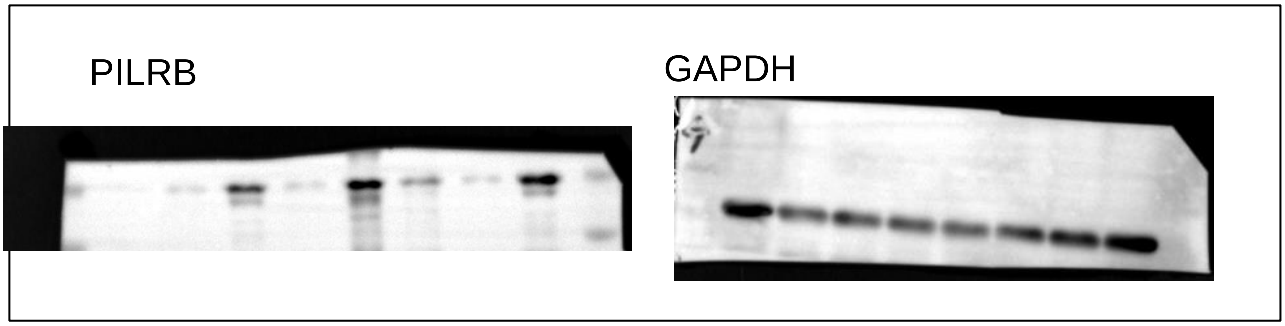

Figure 2B

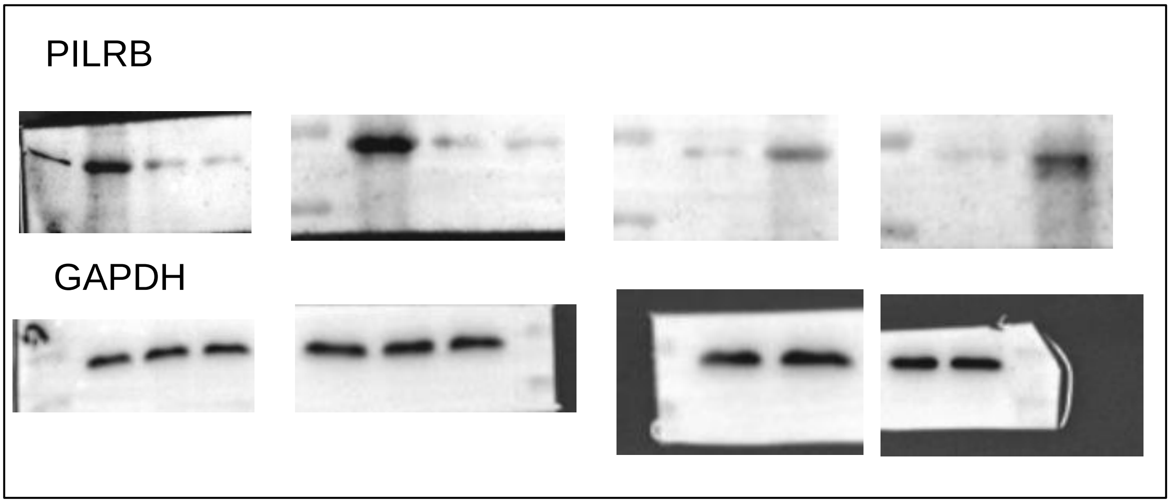

Figure 3B

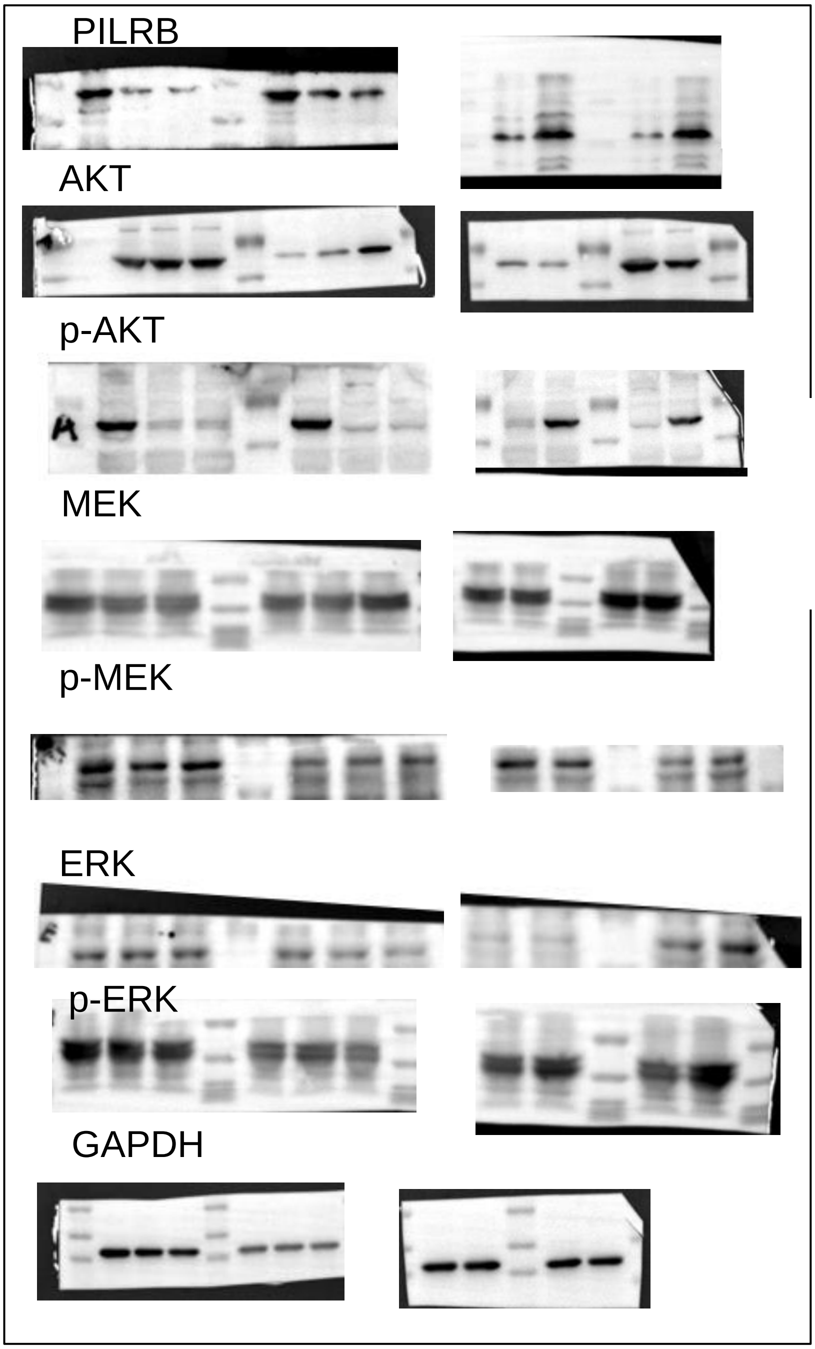

Figure 3C

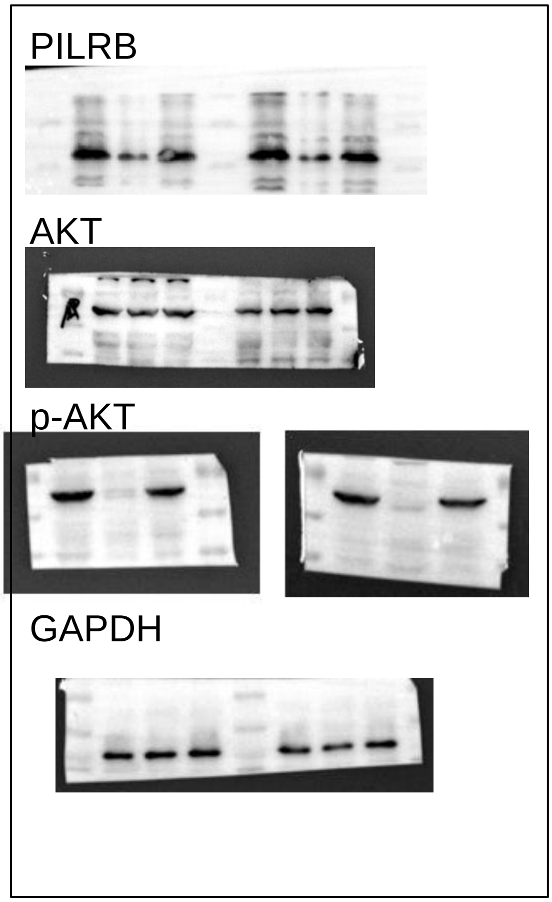

Figure 3D

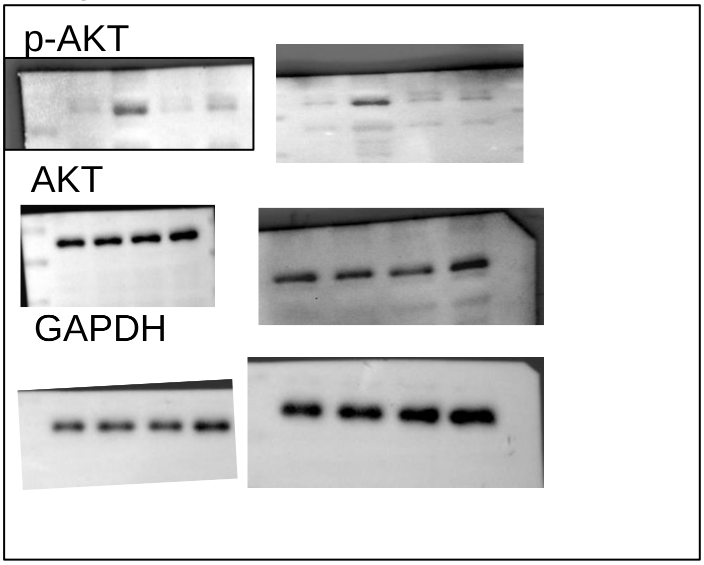

Figure 3F

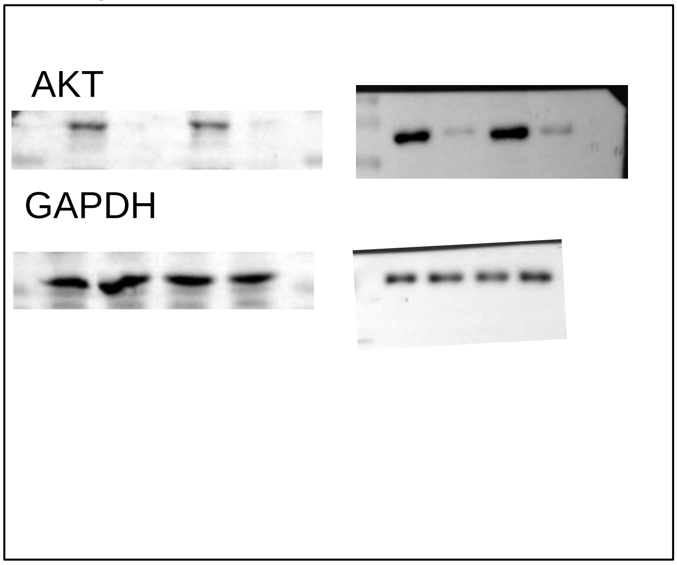

Figure 4A

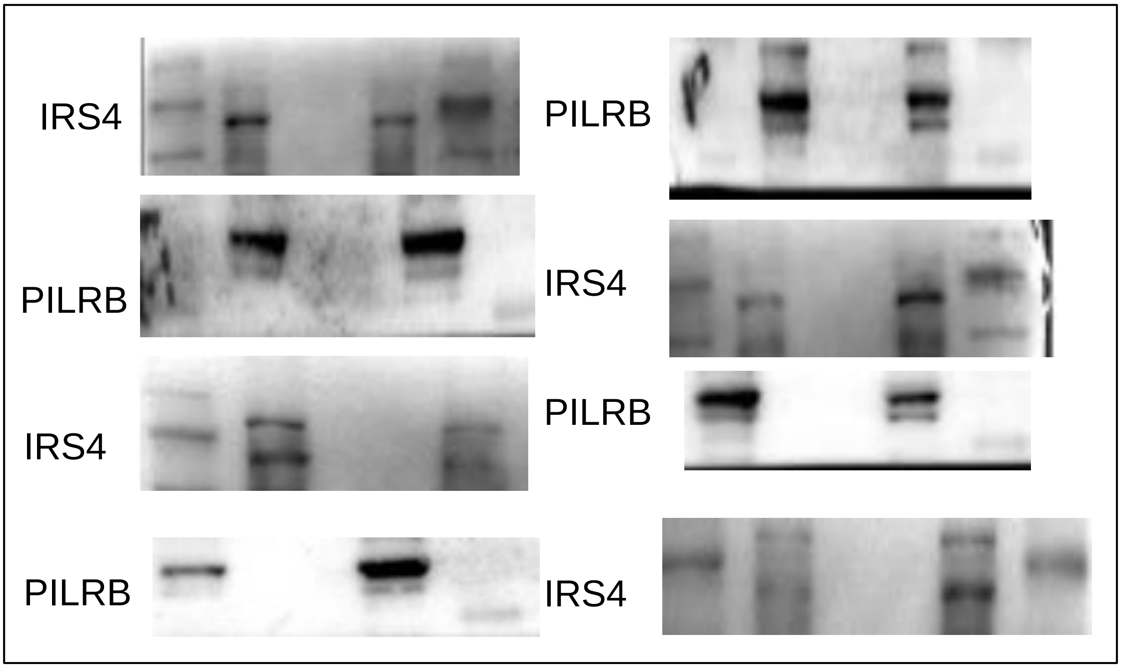

Figure 4C

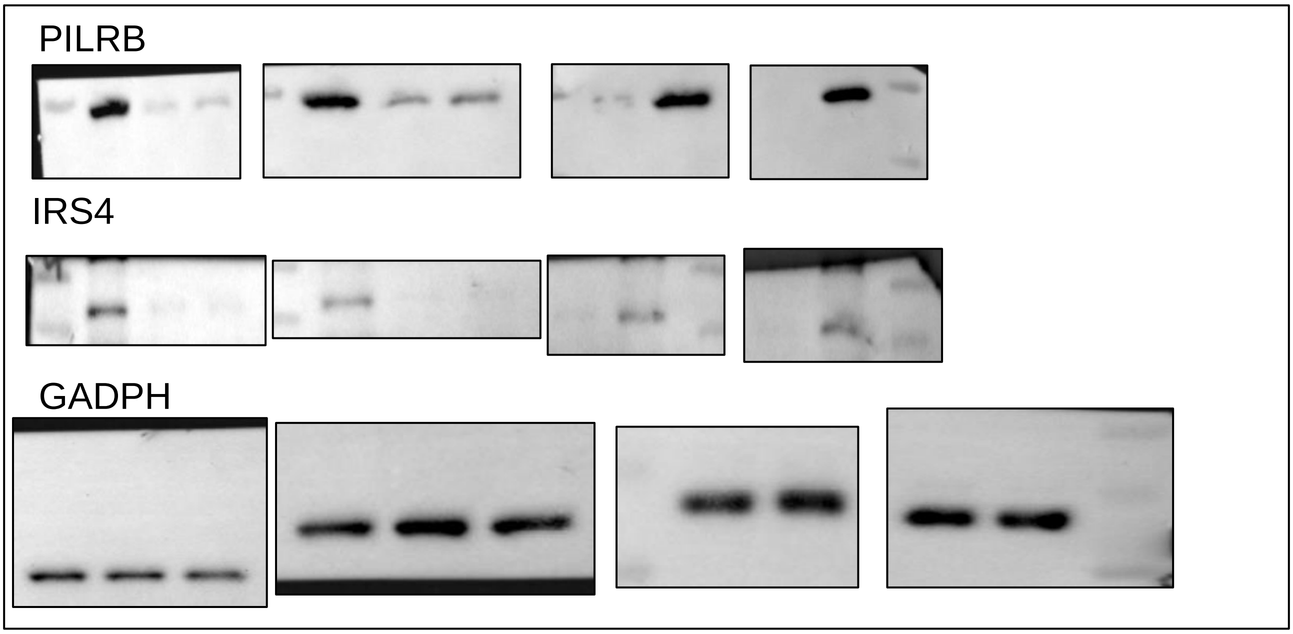

Figure 4D

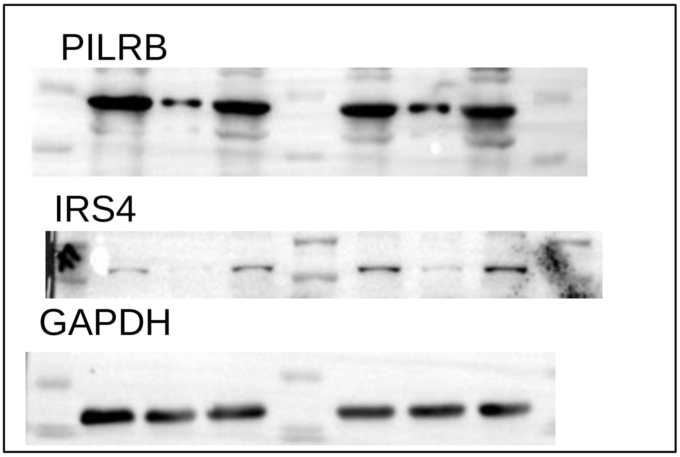

Figure 4I

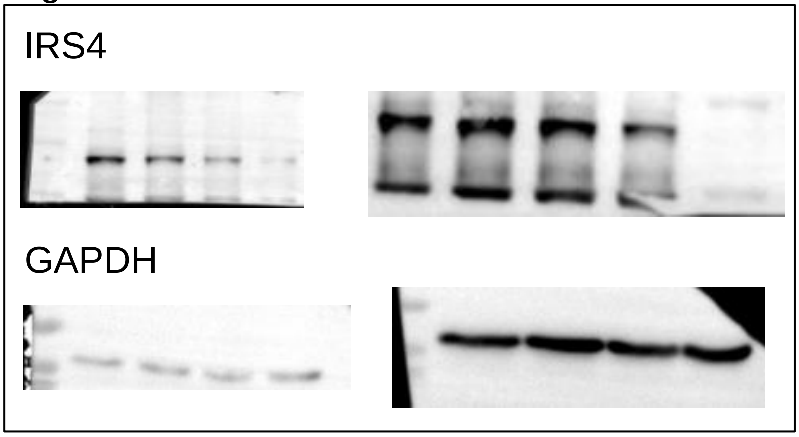

Figure 4J

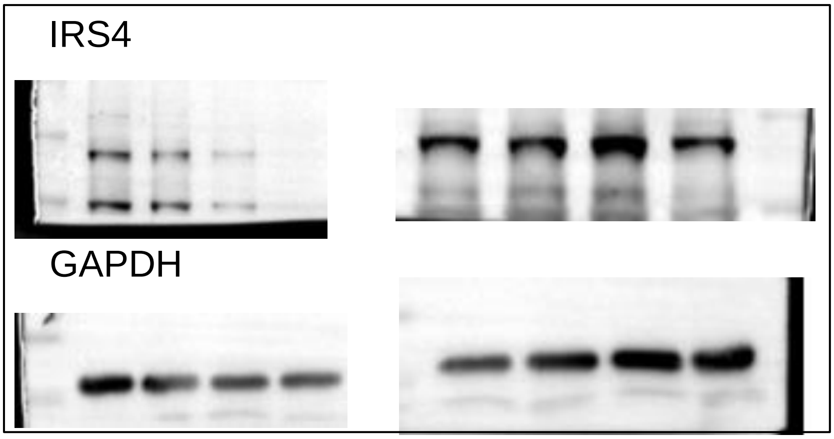

Figure 4M

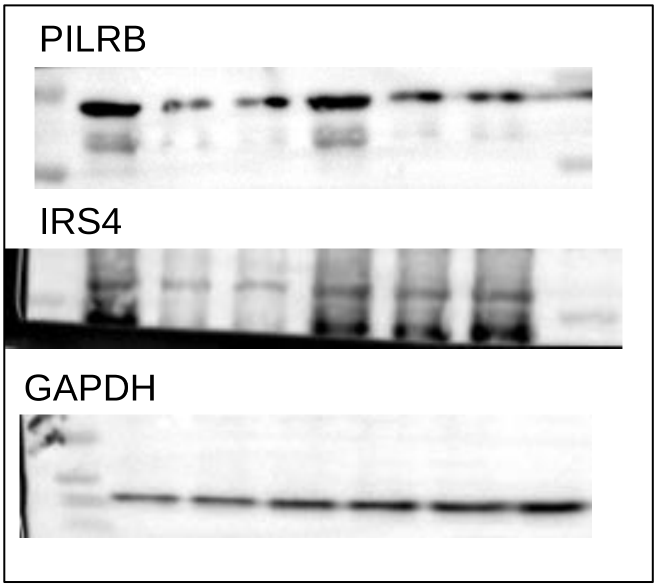

Figure 4N

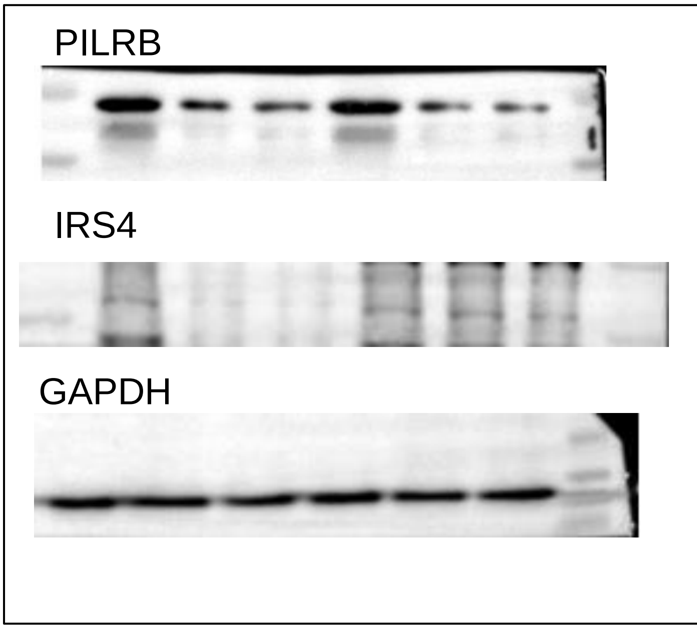

Figure 5A

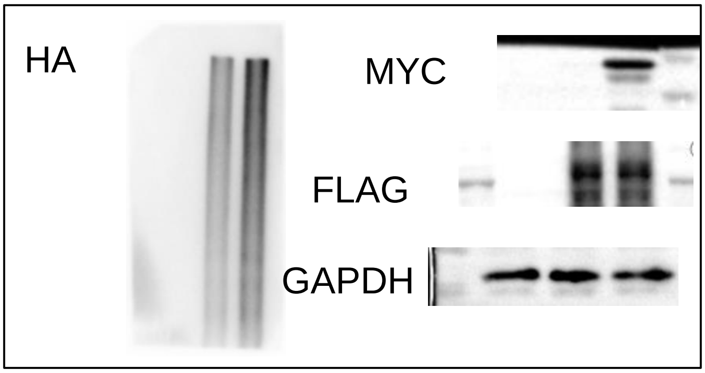

Figure 5B

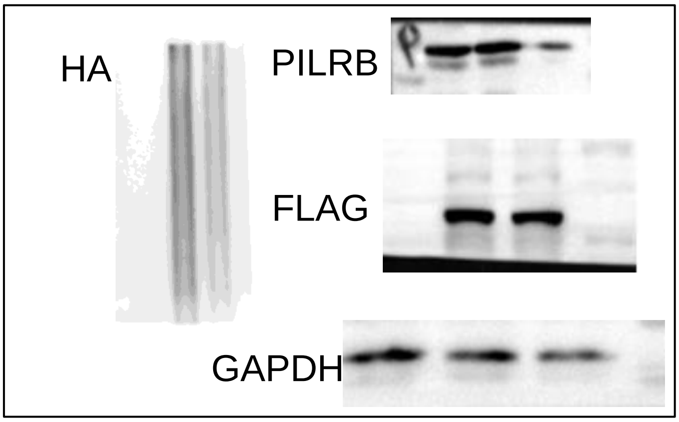

Figure 5C

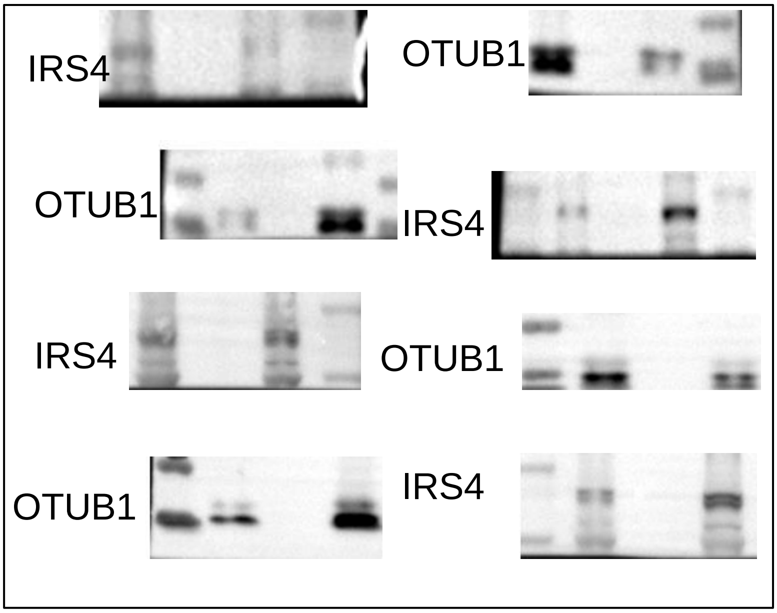

Figure 5D

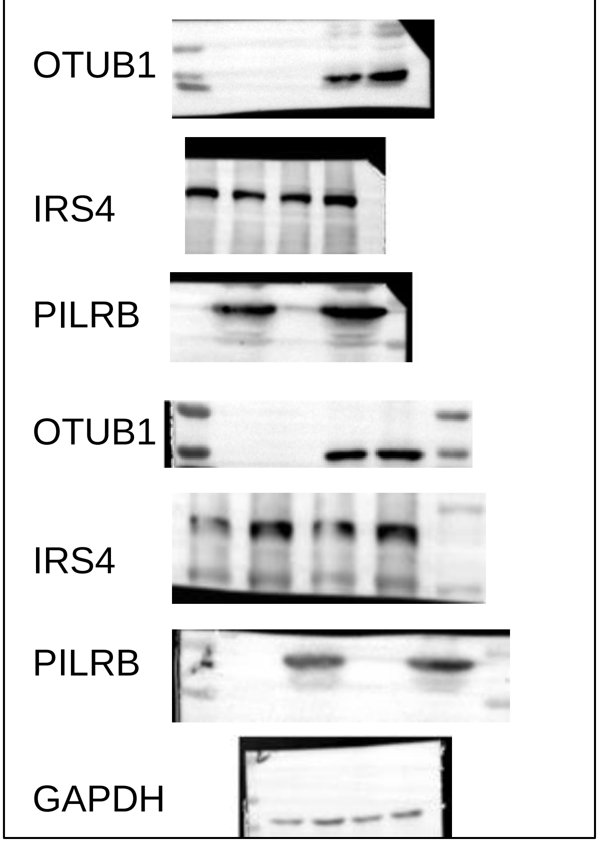

Figure 5E

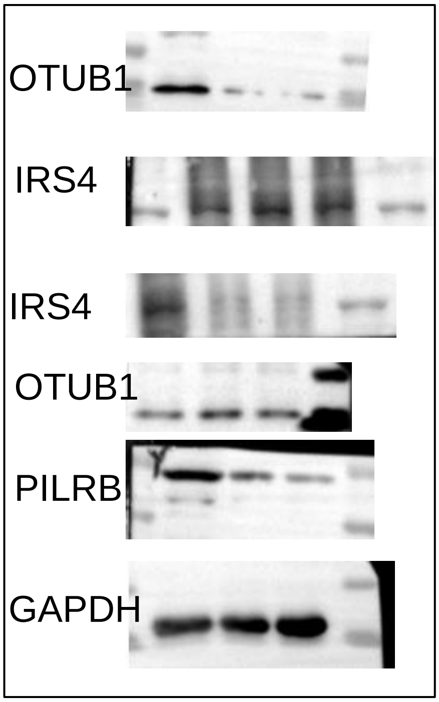

Figure 5F

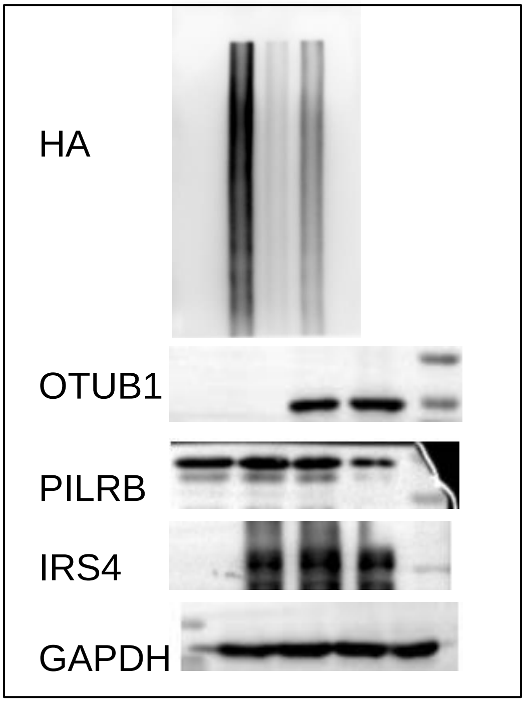

Figure 5G

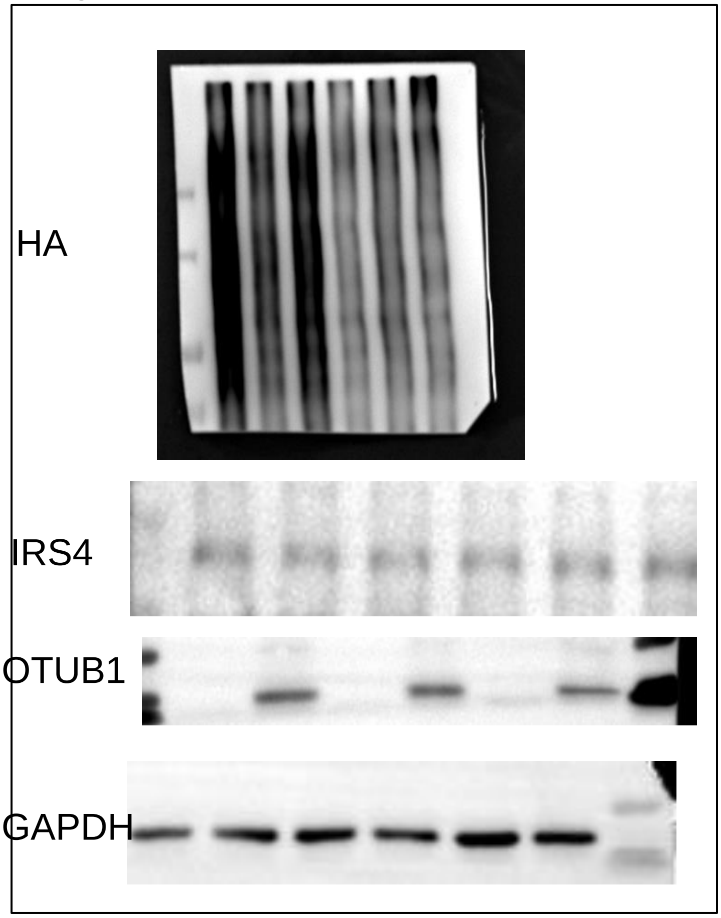

Figure 5H

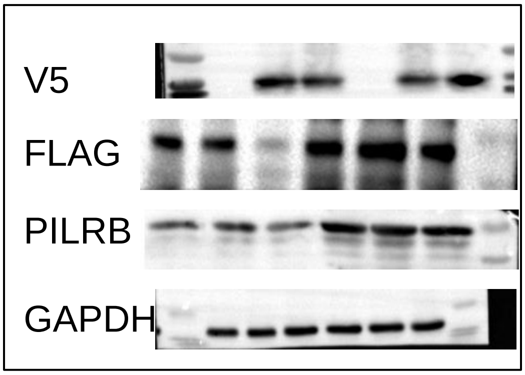

Figure 6A

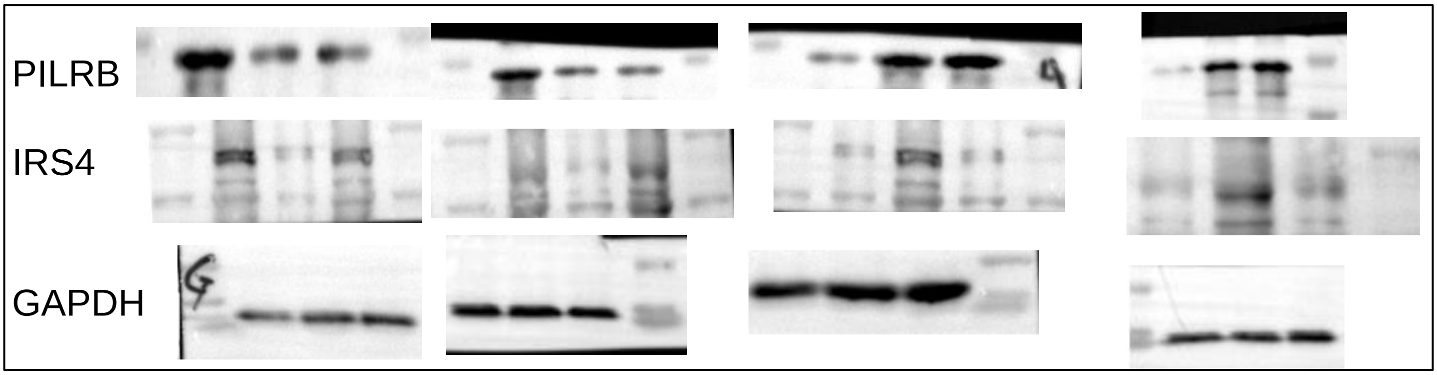

Figure 7C

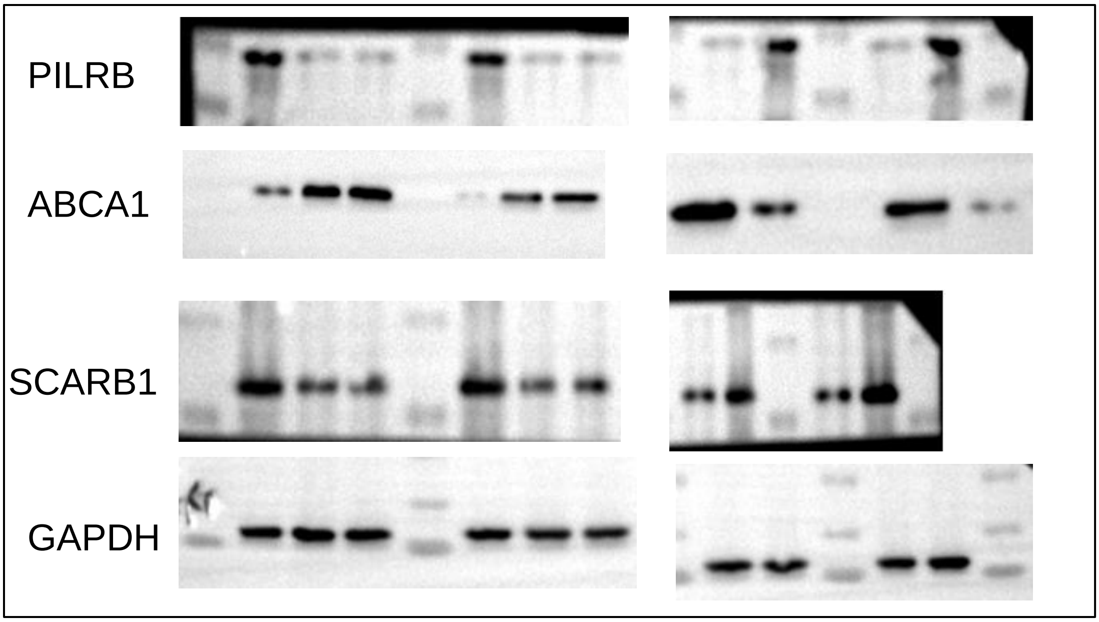

Figure S1

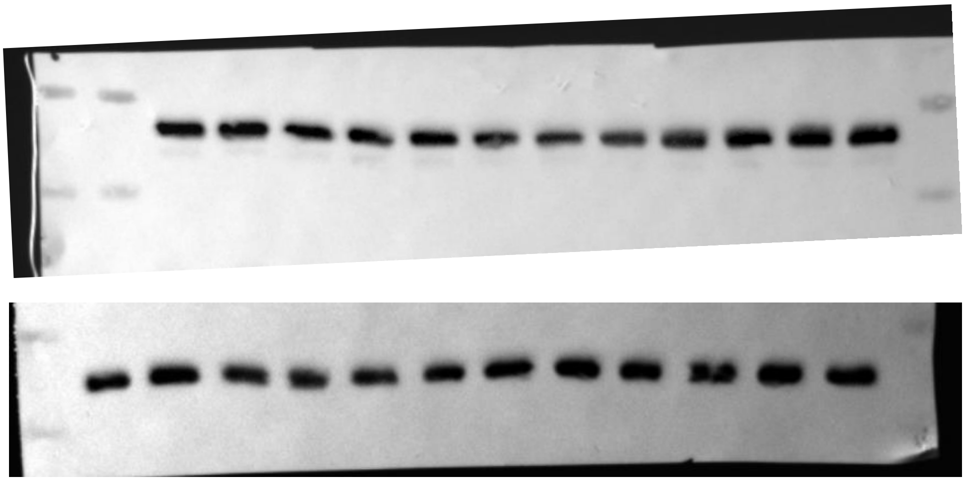

Figure S2B

PILRB

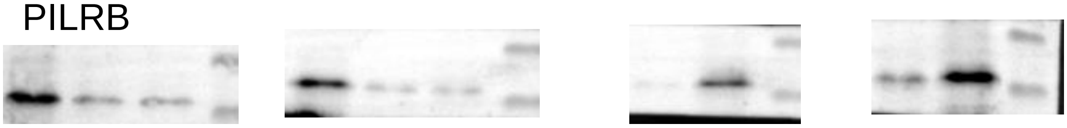

E-cadheein

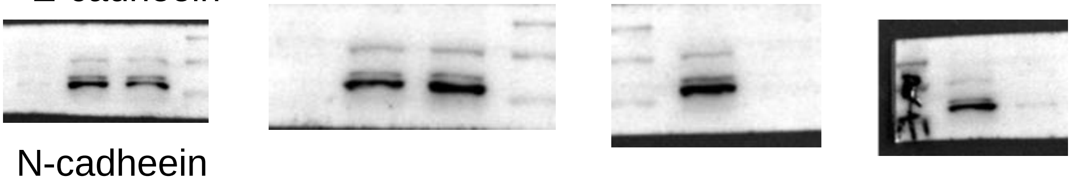

N-cadheein

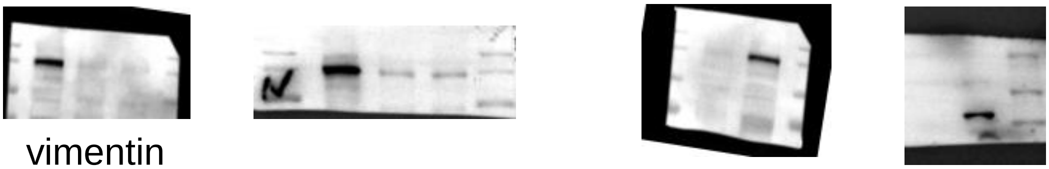

vimentin

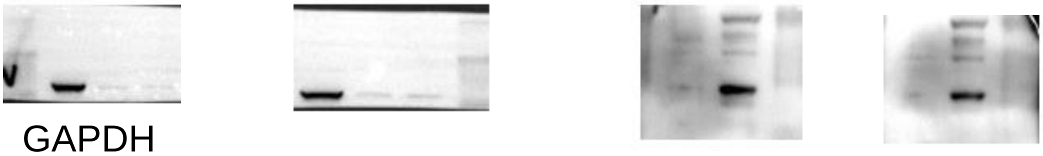

GAPDH

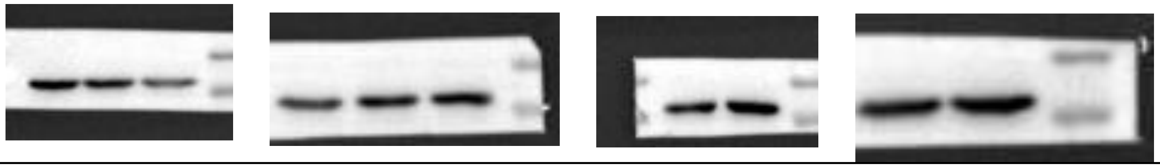

Figure S6A

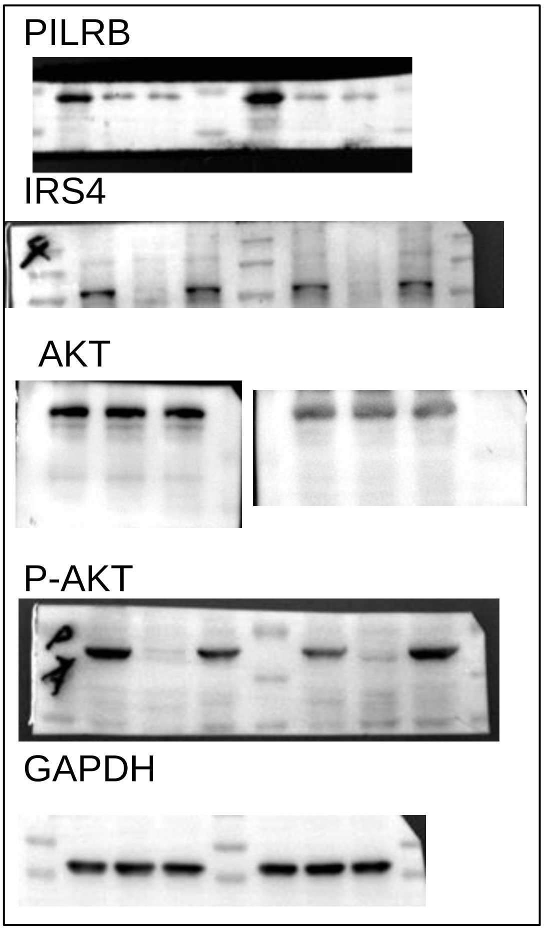

Figure S6C

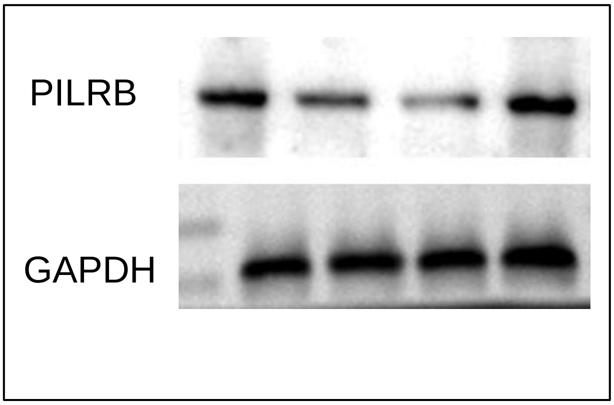

Figure S6B

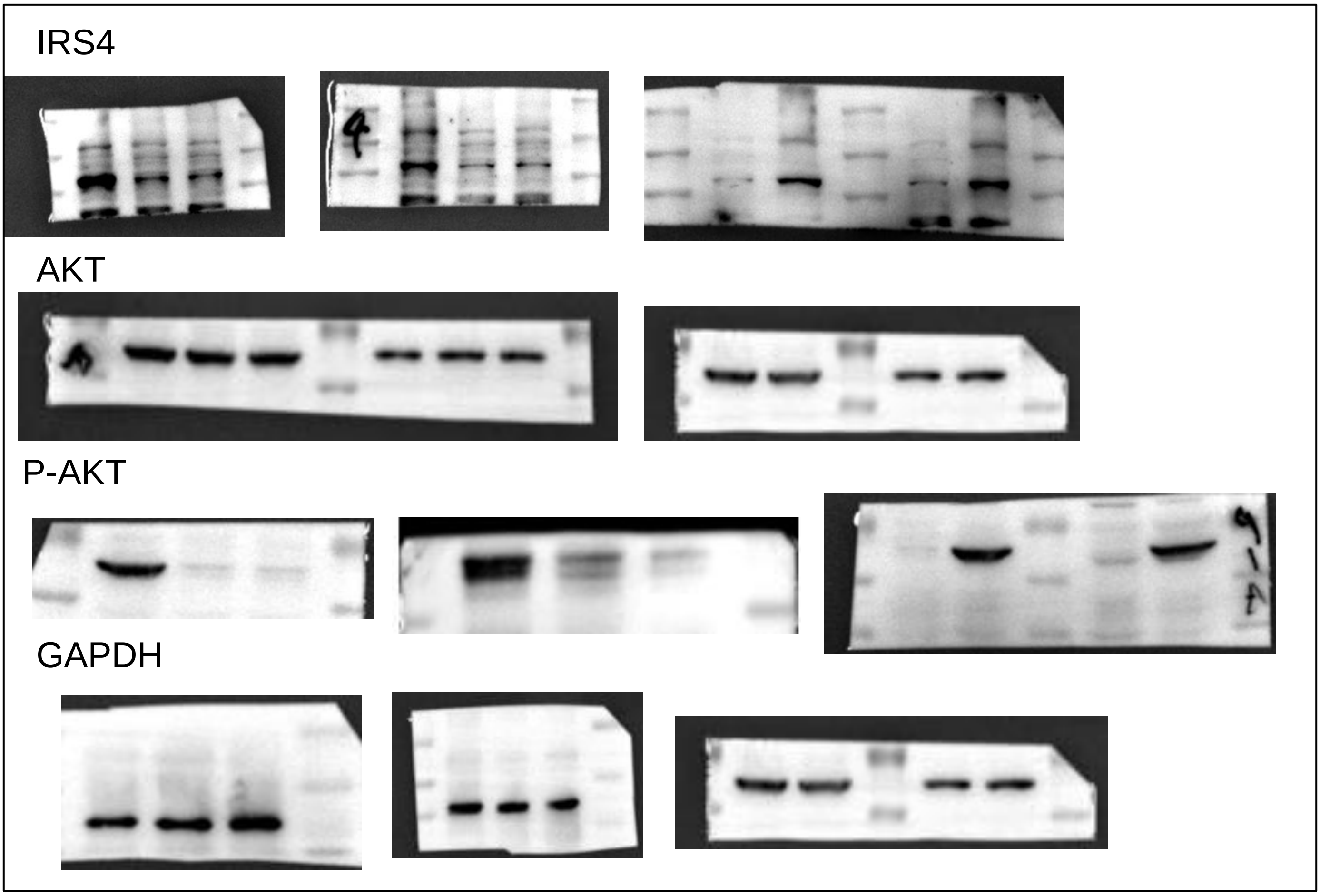

Figure S7B

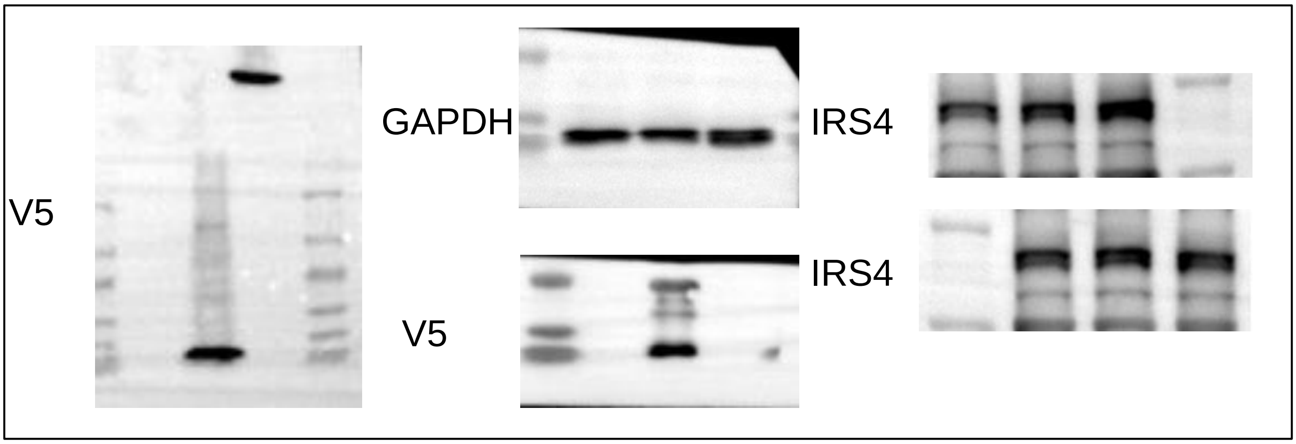

Figure S8C

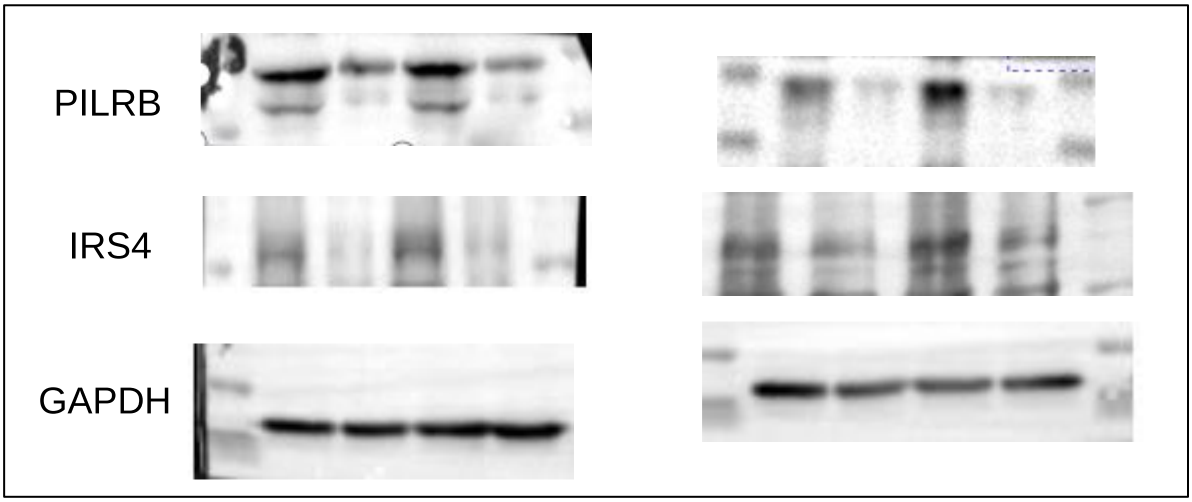

Supplement: Supplementary file 2 — Raw western blot data [file 41419_2024_7026_MOESM2_ESM.pdf]
